# Supplementary material for: Willingness to pay for diabetic retinopathy screening in Qujiang District, rural Guangdong, southern China: a cross-sectional study
Source: BMJ Open. 2023 Apr 25;13(4):e065792. doi: 10.1136/bmjopen-2022-065792 (PMC10151899; doi:10.1136/bmjopen-2022-065792)
Supplement: Author's manuscript [file bmjopen-2022-065792.draft_revisions.pdf]

BMJ Open is committed to open peer review. As part of this commitment we make the peer review history of every article we publish publicly available.

When an article is published we post the peer reviewers' comments and the authors' responses online. We also post the versions of the paper that were used during peer review. These are the versions that the peer review comments apply to.

The versions of the paper that follow are the versions that were submitted during the peer review process. They are not the versions of record or the final published versions. They should not be cited or distributed as the published version of this manuscript.

BMJ Open is an open access journal and the full, final, typeset and author-corrected version of record of the manuscript is available on our site with no access controls, subscription charges or pay-per-view fees (http://bmjopen.bmj.com).

If you have any questions on BMJ Open's open peer review process please email info.bmjopen@bmj.com

BMJ Open

Willingness to Pay for Diabetic Retinopathy Screening in Qujiang District, Rural South China

Journal:	*BMJ Open*
Manuscript ID	bmjopen-2022-065792
Article Type:	Original research
Date Submitted by the Author:	20-Jun-2022

Complete List of Authors:	Xiao, Baixiang; Affiliated Eye Hospital of Nanchang University; Queen's University Belfast,<br>Gu, Xuejun; Affiliated Eye Hospital of Nanchang University<br>Jin, Ling; Sun Yat-Sen University Zhongshan Ophthalmic Center<br>Chan, Ving ; Queen's University Belfast, Center for Public Health<br>Li, Yanping; Nanchang University affiliated Eye Hospital<br>Price-Sanchez, Carlos; Queen’s University Belfast<br>Liu, Yuanping; Sun Yat-Sen University<br>Wang, Yanfang; Zhongshan Ophthalmic Centre, Sun Yat-sen University<br>Fu, Haoxiang; Zhenjiang District Hospital<br>Li, Dongfeng; Queen’s University Belfast<br>Congdon, Nathan; Queen’s University Belfast; Sun Yat-Sen University

Keywords:	Diabetic retinopathy < DIABETES & ENDOCRINOLOGY, EPIDEMIOLOGY, HEALTH ECONOMICS, Health economics < HEALTH SERVICES ADMINISTRATION & MANAGEMENT

SCHOLARONE™ Manuscripts

I, the Submitting Author has the right to grant and does grant on behalf of all authors of the Work (as defined in the below author licence), an exclusive licence and/or a non-exclusive licence for contributions from authors who are: i) UK Crown employees; ii) where BMJ has agreed a CC-BY licence shall apply, and/or iii) in accordance with the terms applicable for US Federal Government officers or employees acting as part of their official duties; on a worldwide, perpetual, irrevocable, royalty-free basis to BMJ Publishing Group Ltd ("BMJ") its licensees and where the relevant Journal is co-owned by BMJ to the co-owners of the Journal, to publish the Work in this journal and any other BMJ products and to exploit all rights, as set out in our licence.

The Submitting Author accepts and understands that any supply made under these terms is made by BMJ to the Submitting Author unless you are acting as an employee on behalf of your employer or a postgraduate student of an affiliated institution which is paying any applicable article publishing charge ("APC") for Open Access articles. Where the Submitting Author wishes to make the Work available on an Open Access basis (and intends to pay the relevant APC), the terms of reuse of such Open Access shall be governed by a Creative Commons licence – details of these licences and which Creative Commons licence will apply to this Work are set out in our licence referred to above.

Other than as permitted in any relevant BMJ Author's Self Archiving Policies, I confirm this Work has not been accepted for publication elsewhere, is not being considered for publication elsewhere and does not duplicate material already published. I confirm all authors consent to publication of this Work and authorise the granting of this licence.

For peer review only

Willingness to Pay for Diabetic Retinopathy Screening in Qujiang District, Rural South China

Baixiang Xiao[1, 2], Xuejun Gu[1], Ling Jin[3], Ving Fai Chan[2,4], Yanping Li[1], Carlos Price-Sanchez[2], Yuanping Liu[3], Yanfang Wang[3], Haoxiang Fu[5], Dongfeng Li[2], Nathan Congdon [2,3,6]

1. Affiliated Eye Hospital of Nanchang University, Nanchang City, China
2. Centre for Public Health, Queen's University Belfast, Belfast, United Kingdom
3. The State Key Laboratory of Ophthalmology, Zhongshan Ophthalmic Center, Sun Yat-sen University, Guangzhou City, China
4. College of Health Sciences, University of KwaZulu Natal. Durban. South Africa
5. Zhenjiang District Hospital, Shaoguan City, Guangdong Province, China
6. Orbis International, NY, United States

Address for correspondence: Prof Nathan Congdon, Centre for Public Health, Royal Victoria Hospital, Queen's University Belfast, 274 Grosvenor Rd, Belfast UK BT12 6BA, and the State Key Laboratory of Ophthalmology, Zhongshan Ophthalmic Center, Sun Yat-sen University, 54 S. Xianlie Road, Guangzhou, People's Republic of China
Email: ncongdon1@gmail.com, Tel: +44 7748751393 (UK) and +86 020 87682342 (P.R.C.)

Keywords: willingness to pay, diabetic retinopathy, screening, rural, China

Word count: 3458

Running head: Willingness to pay for diabetic retinopathy screening
Number of tables: 3
Number of figures: 0

This submission has not been previously published and is not simultaneously being considered by any other publication.

For peer review only

Abstract:

Objectives: To determine the proportion of people with diabetes mellitus (DM) willing to pay and the mean amount willing to pay for a diabetic retinopathy screening (DRS) in Qujiang District of Shaoguan City, rural South China.

Design: This cross-sectional study was conducted through a large scale of DRS in 2019. We randomly selected 575 (21.5%) from among 2677 people over 18 years old with known diabetes who attended the screening. Participants elected to pay nothing or RMB10 – 120 (USD 1.6 – 18.8), in RMB 10 intervals, displayed on printed cards for eye screening. One trained interviewer collected all the data.

Setting: The willingness to pay (WTP) for the DRS study was incorporated as part of the scheduled DRS at 10 primary health centres.

Participants: Five hundred and fourty-five people with diagnosed DM from the 575 randomly selected agreed to participate to the study.

Primary and secondary outcome measures: Proportion of people willing to pay for, the amount people would like to pay for and the factors influenced people to pay.

Results: Among the 545 (94.8%) who took part in the survey (mean age 64.6 years [SD±10.4]), 327 (60.0%) were willing to pay for screening, of whom 273 (83.5%) would pay RMB10 – 30 (USD1.6 – 4.7). People living in rural areas and those from lower-income families were more willing to pay. Among those willing to pay anything, men, urban residents, and those covered by employer-linked insurance were willing to pay larger sums ($p<0.05$ for all).

Conclusions and relevance: Nearly two-thirds of participants were willing to pay for screening in this event organized at the primary level in rural China, which offers the potential that such activities can be sustained and scaled up through user fees.

For peer review only

Strengths and limitations of this study:

- This study was incorporated to actual scheduled large-scale DR screening activities, having been community-based, and supported by the local hospital, health authorities, and primary health staff.
- To determine the sum participants were willing to pay, we used a direct method with payment cards of differing amounts which were presented simultaneously to participants for selection. This avoided starting point bias in bidding for payment.
- To avoid range bias, we used 10 yuan (USD 1.59) for a small scale stable range which would not greatly affect the payment value.
- The study was conducted at a single point and cannot reflect long-term WTP values as the economy is constantly developing. The study also took place in one mostly rural region, so it does not represent other areas in China.

For peer review only

Introduction:

The global prevalence of people living with diabetes mellitus (PwDM) has increased steadily from 108 million in 1980 to 425 million in 2017[1]. It is expected to increase to 693 million cases by 2045[2]. The rate of PwDM in China has followed international trends, with the prevalence of adult PwDM increasing from approximately 2% in the 1980s to over 10% in 2017[3-5]. This dramatic rise in PwDM is due to universal increase in life expectancy, wider adoption of western diets, sedentary lifestyles and expanding urbanization[5]. While DM itself promises to grow as a health concern, complications associated with its progression pose additional risks to health. Diabetes mellitus can lead to blinding ocular complications requiring further ophthalmological care, straining the Chinese public health system. Of these ocular conditions, Diabetic Retinopathy (DR) is the most common and serious, potentially resulting in irreversible blindness. After 20 years, 90% of persons with Type 1 diabetes and 60% with Type 2 will develop some form of retinopathy. A third of these cases will require treatment to prevent irreversible blindness[6]. Given current rates of PwDM and the prevalence of DR, the number of persons with DR in need of laser treatment is estimated to be over 8 million in China at present[5,7].

Interventions at the level of primary care are effective at both reducing the severity and slowing the progression of DR. Methods such as adopting a healthy lifestyle to control blood glucose, adhering to prescribed medicines, undergoing regular retinal examinations, and receiving timely treatment for retinopathy with laser photocoagulation, intravitreal injections, or vitrectomy for more serious cases, have together been proven to reduce blindness from DR by some 90%[8,9]. Regular retinal screening can be effectively promoted or implemented within the

For peer review only

primary care setting. In particular, establishing robust diabetic retinopathy screening (DRS) protocols at the level of primary care is a key step in preventing the progression of DR towards visual impairment (VI) or blindness (BL).

Despite the efficacy of DRS in preventing VI/BL, increasing the uptake of DRS has been a significant challenge for many health systems[10,11]. Common barriers to uptake include: a lack of perceived need, patient fear of positive diagnosis, high cost, lack of access, and low quality of service[10]. Additionally, DR is often asymptomatic before the disease affects the macula or causes a vitreous haemorrhage and begins impairing visual acuity. Therefore, many unscreened patients are unaware they have the condition before it becomes severe[12-17]. As diabetic retinopathy becomes more prevalent, integrating DRS at all levels of service has never been more important.

As part of a pilot study to develop and enhance primary and secondary healthcare DRS programs, Orbis International and the World Diabetes Foundation (WDF) have supported eye care services for PwDM at 6 county hospitals in Guangdong Province since 2017. The current willingness to pay (WTP) study was conducted as part of the DRS program in Qujiang District of Shaoguan City, northern Guangdong Province, in the second half of 2019. Though prior studies in the area have examined WTP for cataract surgery[18], at present, no such studies on DRS have been completed in China. This study aims to fill this gap by exploring WTP for DRS services in rural North Guangdong Province. Despite the current lack of targeted funding for DRS in most areas of China, charging for this crucial service might still be sufficient if beneficiaries are willing to pay enough to sustain the service. The findings in this report will provide context for future decisions regarding providing care for PwDM and establishing of

community DRS services.

Research Design and Methods

Ethical approval for the DRS study in Qujiang, south rural China, was obtained from Zhongshan Ophthalmic Center, Sun Yat-sen University (ZOC) (Approval Number: 2019KYPJ067). Written consent was obtained from each participant. PwDM found with sight-threatening DR (R2 or above on the UKNHS DRS system[19]), operable cataracts, or other suspected eye diseases were referred to secondary-level hospitals for further examination or treatment. The study fulfilled the tenets of the Declaration of Helsinki.

Setting and study participants:

Qujiang is a secondary-level district in China's southern Guangdong province, home to 320,000 people as of 2018 (a population density of 192 people per km^2), with a 2018 per capita gross domestic product (GDP) of USD7,160.32. Qujiang's GDP is significantly less than the national average per capita GDP of USD10,253.97 that same year. These figures are typical of other poor rural areas in Southern China.

Shaoguan City Railway Hospital and Qujiang County Hospital are secondary-level hospitals in Zhenjiang and Qujiang Districts respectively. Two doctors from the eye department of Shaoguan City Railway hospital had been trained in detecting and treating DR and were competent in performing fundus laser treatment and intra-vitreous injection. Trainers for DR screening and managers were also trained in the program. They were then able to arrange

For peer review only - http://bmjopen.bmj.com/site/about/guidelines.xhtml

periodic, small-scale DRS in rural township health facilities and urban community health centers since 2017. Basic examinations for anterior segment eye disease were provided at the outpatient department in Qujiang Hospital. Patients suspected of having posterior segment diseases, including DR, would have to travel to the neighbouring district hospital in Zhenjiang or a facility in the city centre.

As with many other places in China, persons diagnosed with diabetes are encouraged to register with the primary health system in Qujiang District for a free annual health check, monitoring of complications, and health education. Village doctors (VDs) provide free fasting blood glucose checks for people aged over 35 years or anyone wishing to test their glucose levels. People suspected of having DM are referred for further examination at secondary-level hospitals. A total of 3,646 PwDM registered across 10 primary health centres in Qujiang District were informed of these local health bureau-supported DRS services, at which point they were able to schedule appointments with trained VDs.

Study team members at the screening sites included an ophthalmologist, two nurses, one technician, and one interviewer, all trained for the study. PwDM came to the township health units for the screening at a pre-scheduled time.

Of the 3,646 PwDM informed about the screening, 2,677 (73.4%) from across the 10 towns in Qujiang attended. This was the first formally arranged, large-scale DRS conducted in the district. We randomly approached 575 PwDM attending the DRS by generating a random number for the selection of a first participant to the WTP study and then approaching every sixth participant from that point on who arrived for screening. In total, 545 (94.8%) agreed to

For peer review only

participate in the WTP study and were recruited.

Patient and public involvement statement:

All the participants were informed of the scheduled DRS 1-2 months before screening started, through the trained village doctors in the study area and posters circulated in the villages. They were explained of the willingness to pay study by the interviewers at the screening site. Research questions were developed upon medical practice and pricing which authors were familiar with as they mostly were local medical staff. Participants were not involved in the designing of the study directly although the draft questionnaires were tested with some PwDM previously.

Data collection:

Participants were welcomed to the scheduled annual DR screening by their local public health doctor at each screening site. The local physician would then personally inform each participant once their screening report was available via mobile phone, text, or WeChat message within one week. Participants were then supplied with DR educational bulletins and posters, which provided information on DR and highlighted the importance of receiving annual fundus imaging for the early detection of ocular diseases.

Participants' basic information and disease history were collected and double-checked via the Chronic Disease Recording System. Retrieved data included participant name, age, sex, site of residence (rural or urban), education, profession, insurance, self-assessment of visual acuity, economic situation upon the proportion of family income spent on food, and history of diseases including DM and hypertension. Venous blood was collected from each participant to monitor

For peer review only

HbA1c upon the National Chronic Disease Management Guideline. Presenting visual acuity (PVA) was tested monocularly in each eye at 6 meters with a retro-illuminated Snellen visual chart before the ophthalmologist carried out a slit lamp examination. Intraocular pressure (IOP) was measured in each eye using tono-pen before pupils were dilated with compound tropicamide to enable the capture of fundus images with Canon CR-2 by a trained nurse. Patients with high IOP or shallow anterior chambers detected under slit lamp examination were not dilated; instead, images were taken through pupils which had been dilated scotopically after around 3 to 5 minutes in a darkened room.

Diabetic complications were defined as any of the following symptoms: ulcers on hands or feet, cardiovascular disease, diabetic nephropathy, diabetic eye disease, ketoacidosis or other serious metabolic complications. Participants could self-assess their physical health and visual acuity on a scale of 1-5, corresponding to: bad (1), not good (2), alright (3), good (4) and excellent (5). Regarding their economic situation, respondents were given two choices related to the proportion of monthly family income spent on food: less than 30%, equal to or over 30%.

Questionnaires regarding WTP for the DR screening were administered post screening in a quiet, separate room. Each participant was given the opportunity to ask any questions they might have on DM, DR, screening, or the report, and received informed responses from the trained interviewer. Comments on the screening were also invited. The participant was then told that the screening required appropriate human resources, equipment, and preparation, so there was some cost. The direct cost (travel or transport cost, daily allowance for screening staff) of one person screened was approximately USD4.7. At the time of the study, there was

For peer review only

no budget in the primary health care system for the DRS. According to the provincial pricing regulations, fundus imaging charges USD 18.8 for both eyes at secondary level hospitals. Ultimately these costs fall on the patient, as expenses associated with imaging and registration are not currently reimbursed by insurance.

Each participant was informed that their choice of willingness to pay category would not interfere with their ability to receive primary health care, medical services in hospitals, nor current or any future DR screening. Participants were first asked whether they would like to pay something, starting at 10 yuan (USD 1.6), for the screening. Reasons were elicited for those answering "no". When a participant expressed willingness to pay any amount, they were presented with cards marked from 10 yuan (USD 1.6) to 120 yuan (USD 18.8) at intervals of 10 yuan (USD 1.6) (12 cards in total). To avoid bargaining bias, we presented subjects with cards valued at RMB10, 20, 30, 40, 50, 60, 70, 80, 90, 100, 110 and 120 simultaneously for their selection. A trained interviewer collected all data to maintain the consistency and quality of the interview.

Statistical analysis:

Participants' characteristics were presented as mean (standardised deviation, SD) for continuous data with normal distribution or median (inter quartile range, IQR) for data without normal distribution, and frequency (%) for categorical variables. Shapiro–Wilk normality test and histogram were applied to check the normality of continuous data. Participants' age was categorised by an interval of 10 years. Duration of diabetes was categorized by interval of 5 years. The cut-offs of 6/60, 6/18 and 6/12 were used to describe visual acuity in better-seeing

For peer review only

eye.

Logistic regression analysis for Willingness to Pay and linear regression analysis for the amount of people willing to pay were employed to examine the associations between potential factors and outcomes. Univariable and multivariable regression models were fitted. Age, sex and variables with $P<0.20$ in the univariable regression analysis were included in the multivariable regression model. All statistical analyses were performed using a commercially available software package (Stata 16, Stata Corp, College Station TX, USA).

Results:

Among 545 participants, the mean age was 64.4±10.4 years (range 35-92 years), 323 [59.3%] were women. (Table 1) A total of 345 (63.3%) participants lived in urban settings and 200 (36.7%) in rural areas. Half (44.6%) of participants had received a primary school education, 54 (9.9%) attended high school or above, and 81 (14.9%) had never received any formal education. All participants had either normal resident medical insurance (n=372, 68.3%) or employment-linked insurance (n=173, 31.7%).

For peer review only

Table 1. Demographic characteristic of participants to the willingness to pay study, Qujiang, China

Characteristic	Mean (SD)/n (%)
Total	545
**Age (year), mean (SD)**	64.6 (10.4)
<50	48 (8.8)
50-59	131(24.0)
60-69	198(36.3)
70-79	137(25.1)
80+	31(5.7)
**Gender, n (%)**	
Male	222 (40.7)
Female	323 (59.3)
**Duration of DM (year), n (%)**	
<5	279 (51.2)
5-10	130 (23.9)
10-15	73 (13.4)
>15	63 (11.6)
**Whether with complications, n (%)**	
Yes	434 (79.6)
No	111 (20.4)
**Place of living, n (%)**	
Urban (town) residents	345 (63.3)
Rural (countryside) residents	200 (36.7)
**Education, n (%)**	
Never received formal education	81 (14.9)
Primary school	243 (44.6)
Junior high school	167 (30.6)
High school or above	54 (9.9)
**Presenting visual acuity in better-seeing eye, n (%)**	
>=6/12	435 (79.8)
>/=6/18 to <6/12	72 (13.2)
>/=6/60 to <6/18	32 (5.9)
<6/60	6 (1.1)
**Medical insurance, n (%)**	
Resident medical insurance	372 (68.3)
Employment-linked insurance	173 (31.7)
**Self-assessment of current health situation, n (%)**	
Bad	11 (2.02)
Not good	107 (19.6)
Good	261 (47.9)
Very good	153 (28.1)
Excellent	13 (2.39)
**Self-assessment of current visual acuity, n (%)**	
Bad	12 (2.20)
Not good	101 (18.5)
Good	349 (64.0)
Very good	74 (13.6)
Excellent	9 (1.65)
**Economic situation, n (%)**	
<30% of monthly family income spent on food	108 (19.8)
⩾30% of monthly family income spent on food	437 (80.2)

SD: Standard deviation

For peer review only

Approximately half (51.2%) of participants had been diagnosed with DM within the last 5 years, around one-fourth (23.9%) within 5-10 years, and only 63 (11.6%) had been diagnosed over 15 years prior (Table 1). Most participants (n=435, 79.8%) had PVA >= 6/12 and only 6 (1.1%) were < 6/60 in the better-seeing eye. Most participants (n=434, 79.6%) did not report any diabetic complications. Nearly 80% of participants reported being in good, very good, or excellent health at the time of the screening, and a similar proportion described their vision as falling in the same three categories. Regarding their economic situation, most participants (80.2%) reported their households spent 30% or above of their monthly family income on food, while 19.8% spent less than 30%. (Table 1)

Overall, 327 (60%) participants expressed a willingness to pay for the DRS at the primary level, of whom 273 (83.5%) would pay RMB10 – 30 (USD 1.6 – 4.7), 34 (10.4%) would pay RMB 40 – 50 (USD 6.3 – 7.8), and 15 (4.6%) would pay RMB 80 -120 (USD 12.5 – 18.8). In logistic regression models, only rural residence and paying =/> 30% of income for food (both more willing) were associated with WTP anything for DRS (Table 2). Both variables were significant in the univariate and multivariate models.

Among 327 PwDM who were willing to pay something for screening, in univariate linear regression models, men, urban residents, and those with higher educational levels and covered by employment-linked insurance were willing to pay more (Table 3). Conversely, participants with worse self-rated health and vision were willing to pay less. Only the associations with sex, urban residence and health insurance remained significant in the multivariate models.

For peer review only

Table 2. Logistic Analysis of Willingness to Pay for Diabetic Retinopathy Screening, Qujiang, China (n=545)

Characteristic	Univariable regression		Multivariable regression* (n=545)	
	OR (95% CI)	P	OR (95% CI)	P
Age (per year)	0.99 (0.97, 1.01)	0.217	1.00 (0.98, 1.02)	0.690
Female	0.83 (0.59, 1.18)	0.302	0.98 (0.66, 1.44)	0.903
Duration of DM (year)				
<5	Reference		Reference	
5-10	0.67 (0.44, 1.03)	0.066	0.71 (0.46, 1.11)	0.138
10-15	0.64 (0.38, 1.08)	0.094	0.75 (0.43, 1.30)	0.306
>15	0.85 (0.48, 1.49)	0.568	1.18 (0.64, 2.19)	0.591
Complications	0.89 (0.58, 1.37)	0.602		
Place of living				
Rural residents	Reference		Reference	
Urban residents	0.46 (0.31, 0.66)	<0.001	0.44 (0.28, 0.69)	<0.001
Education				
Never received formal education	Reference		Reference	
Primary school	1.07 (0.64, 1.78)	0.795	1.31 (0.75, 2.28	0.350
Junior high school	1.16 (0.68, 1.99)	0.579	1.39 (0.74, 2.63	0.310
High school or above	1.81 (0.87, 3.75)	0.113	2.07 (0.90, 4.78	0.087
Presenting visual acuity in better-seeing eye				
>/=6/12	Reference			
>/=6/18 to <6/12	0.93 (0.51, 1.71)	0.823		
>/=6/60 to <6/18	1.03 (0.58, 1.82)	0.918		
<6/60	1.33 (0.24, 7.36)	0.741		
Medical insurance				
Employment-linked insurance	Reference		Reference	
Normal resident insurance	1.41 (0.98, 2.03)	0.066	0.94 (0.59, 1.51)	0.804
Self-assessment of health				
Bad	Reference			
Not good	0.96 (0.26, 3.47)	0.947		
Good	0.70 (0.20, 2.46)	0.582		
Very good	1.02 (0.29, 3.63)	0.978		
Excellent	3.14 (0.45, 22.0)	0.248		
Self-assessment of visual acuity				

For peer review only

Bad	Reference		Reference	
Not good	1.00 (0.30, 3.38)	0.996	1.09 (0.30, 3.92)	0.894
Good	0.96 (0.30, 3.08)	0.944	0.74 (0.21, 2.55)	0.629
Very good	1.80 (0.51, 6.32)	0.357	1.13 (0.29, 4.37)	0.854
Excellent	5.71 (0.53, 61.4)	0.150	4.03 (0.34, 47.5)	0.268
Economic situation				
<30% monthly income on food	Reference		Reference	
⩾30% on food	1.66 (1.09, 2.54)	0.018	1.70 (1.04, 2.77)	0.035

OR: Odds ratio, CI: Confidence interval

* Age, sex and variables with P<0.20 in the univariable regression analysis were included in the multivariable regression model.

For peer review only

Table 3. Linear Regression of Subjects Willing to Pay Something for Diabetic Retinopathy Screening, Qujiang, China (N=327) *

Characteristic	Univariable regression		Multivariable regression† (n=327)	
	□ (95% CI)	P	□ (95% CI)	P
Age (per year)	-0.0003 (-0.003, 0.002)	0.847	-0.002 (-0.004, 0.001)	0.281
Women	-0.10 (-0.16, -0.05)	<0.001	-0.07 (-0.13, -0.02)	0.013
Duration of DM (year)				
<5	Reference			
5-10	0.02 (-0.05, 0.09)	0.628		
10-15	0.006 (-0.08, 0.10)	0.890		
>15	0.05 (-0.04, 0.14)	0.283		
Complications	0.04 (-0.03, 0.11)	0.300		
Place of living				
Rural residents	Reference		Reference	
Urban residents	0.16 (0.10, 0.21)	<0.001	0.14 (0.07, 0.20)	<0.001
Education				
Never received formal education	Reference		Reference	
Primary school	0.15 (0.06, 0.23)	0.001	0.06 (-0.02, 0.14)	0.162
Junior high school	0.13 (0.04, 0.22)	0.005	0.0001 (-0.10, 0.10)	0.998
High school or above	0.22 (0.11, 0.33)	<0.001	0.09 (-0.03, 0.20)	0.149
Presenting visual acuity in better-seeing eye				
>/=6/12	Reference		Reference	
>/=6/18 to <6/12	-0.04 (-0.14, 0.07)	0.496	-0.005 (-0.10, 0.09)	0.911
>/=6/60 to <6/18	-0.08 (-0.18, 0.01)	0.080	-0.02 (-0.11, 0.07)	0.707
<6/60	0.05 (-0.21, 0.30)	0.718	0.17 (-0.08, 0.42)	0.173
Medical insurance				
Employment-linked insurance	Reference		Reference	
Normal resident insurance	-0.15 (-0.21, -0.09)	<0.001	-0.08 (-0.15, -0.004)	0.040
Self-assessment of health				
Bad	Reference		Reference	
Not good	-0.27 (-0.47, -0.07)	0.009	-0.13 (-0.47, 0.21)	0.455
Good	-0.22 (-0.42, -0.02)	0.028	-0.07 (-0.43, 0.28)	0.696
Very good	-0.26 (-0.46, -0.06)	0.010	-0.08 (-0.43, 0.28)	0.672
Excellent	-0.22 (-0.47, 0.02)	0.073	-0.32 (-0.73, 0.10)	0.134
Self-assessment of VA				
Bad	Reference		Reference	
Not good	-0.28 (-0.48, -0.08)	0.007	-0.11 (-0.46, 0.24)	0.532
Good	-0.23 (-0.43, -0.04)	0.018	-0.05 (-0.41, 0.31)	0.781
Very good	-0.27 (-0.47, -0.07)	0.008	-0.06 (-0.42, 0.31)	0.756
Excellent	-0.03 (-0.29, 0.23)	0.835	0.33 (-0.10, 0.76)	0.135
Economic situation				
<30%	Reference			
⩾30%	0.007 (-0.07, 0.08)	0.847		

□: Parameter coefficient estimate, CI: Confidence interval

* The amount of people willing to pay was not normally distributed and logarithmic transformation was applied.

† Age, sex and variables with P<0.20 in the univariable regression analysis were included in the multivariable regression model.

For peer review only

Discussion:

In this study, 545 randomly selected people with diagnosed diabetes who attended the first

large scale, primary care diabetic retinopathy screening in rural south China were interviewed. Nearly two-thirds (60%) of participants were willing to pay anything for diabetic retinopathy screening. The groups most likely to be willing to pay were rural-dwellers and economically less-advantaged families. Among participants willing to pay anything, those willing to pay more included men, urban-dwellers, and those covered by employment-linked insurance. This study has highlighted a considerable willingness to pay for these services among primary care patients.

The only other WTP study for eye health services in China, conducted in 2006 on cataract surgeries, established that 73% of cataract patients were willing to pay something, and that many were willing to pay more for experienced surgeons and higher-quality intra-ocular lenses[20]. Possible reasons for a lower proportion willing to pay anything for DRS as compared to cataract might include the belief that DRS is a public health service and therefore should be paid for by the government; cataract surgeries are in most cases associated with significant improvements in vision; and coverage rates of medical insurance have increased in China since 2008, potentially reducing willingness to pay out of pocket for healthcare services.

Many countries have experimented with DR screening models to reduce barriers to access and increase service uptake. For example, the United Kingdom and some other European countries have established free community screening for DR[21,22] and found it to be cost-effective[23]. Tele-ophthalmology and general practitioners have been used in the United States, with services either reimbursed by insurance, or paid fully or in part out-of-pocket[24]. General practitioners' referrals for those detected at DR screenings to retinal specialists for further treatment were shown to be effective in preventing VI from DR in Australia[25,26]. There are also established

For peer review only

DR screening services in Asian countries such as Singapore[27], India[27,28] and Taiwan[29].

This study suggests that rural residents and those who are less economically advantaged are willing to pay more for DRS services. In Qujiang District, Shaoguan City, rural residents with DM would otherwise have to travel to the neighboring district hospital or the city centre to seek eye services. Given that there is no public transportation, this trip would take over an hour. Traveling by motorcycle was the most common mode used by rural residents, making an hour-long trip difficult. Additionally, many elderly could only be taken to the city or the neighboring district with the help of a younger person. The fact that this program increased ease of access by bringing DRS services into rural areas may explain why rural residents were more likely to be willing to pay for screening at their township health unit.

Our study found that people covered by employment-linked insurance were more likely to pay higher fees for DRS. This is likely due to their having comparatively better medical insurance and possibly higher incomes. Unfortunately, it was not possible to collect information on income to test this hypothesis in the current study.

In the current study, men and urban residents were willing to pay more for DRS. This could potentially be explained by their being more economically advantaged on average, or their having greater awareness of the importance of DRS. Urban residents may be more apt to understand the need for examinations to prevent diabetic complications and this suggests that future health education interventions should target rural residents, especially rural women.

Another study has reported that more severe DR among persons with type 2 diabetes was

For peer review only

associated with lower perceived utility from the patient's preference-based viewpoint[30]. We did not analyse this metric as the number of patients at R3 was not great enough (9 people, 1.7% among the 545 participants). Rather, we compared the self-assessment of visual acuity and WTP, but found no association between WTP and self-assessed health or vision.

Strengths of the current study include its relevance to actual screening activities, having been community-based, and incorporated into a scheduled, large-scale DR screening program supported by the local hospital, health authorities, and primary level health staff. Additionally, to determine the sum participants were willing to pay, we used a direct method with payment cards of differing amounts which were presented simultaneously to participants for selection. This avoided starting point bias in bidding for payment. To avoid range bias[31], we used 10 yuan (USD 1.59) for a small scale stable range which would not greatly affect the payment value. The minimum value of 10 yuan is equal to the common consultant fee for a hospital outpatient department, and the maximum value of 120 yuan (USD 19.01) is equal to the sum charged for two-eye fundus images taken at secondary level hospitals. The presented value on payment cards was empirical and conceptual, taking account of the actual pricing of ocular medical services as well as the inherent monetary value of 10 yuan (GBP1.2).

Weaknesses must also be acknowledged: The study was conducted at a single point and cannot reflect long-term WTP values as the economy is constantly developing. The study also took place in one mostly rural region, so it does not represent other areas in China. This WTP study is hypothetical and assumes that screening is beneficial to the health of participants. Cost of screening was introduced and reinforced by eliciting payment amounts. We have also assumed that the respondents were willing to accept the screening as a benefit to their health. The

For peer review only

amounts which participants reported they were willing to pay and the amounts they would actually pay may not be the same[31].

To our knowledge, this is the first study on WTP for DRS in China. Cost has been highlighted as the major barrier to establishing DRS at the primary level in China, outside of wealthy areas such as Shanghai, where DRS is well established and fully covered by the metropolitan city government. Findings from this WTP study provide evidence that substantial screening costs could potentially be covered for by patients.

While a considerable percentage of interviewed PwDM demonstrated willingness to take on some personal cost for DRS, studies have noted that organised screening programs result in substantial cost savings for national health systems[19,30]. Recognising that some 50% of current national medical expenditures in China are covered by government medical insurance, investment to establish systematic DRS at the primary care level could substantially reduce costs for the national healthcare system, as well as reducing out-of-pocket medical expenditures[19]. Conducting additional studies in urban areas, as well as in other diverse settings, would contribute to a more complete picture of WTP for DRS in China and help guide the establishment of organised DRS programs.

Conclusions:

Nearly two-thirds of participants were willing to pay for screening in this event organized at the primary level in rural China, which offers the potential that such activities can be sustained and scaled up through user fees.

For peer review only

Contributorship statement:

Baixiang Xiao: Designed and monitored the whole study, initially drafted and revised the manuscript.

Ling Jin: Analyzed data.

Xuejun Gu, Ving Fai Chan, Yanping Li, Dongfeng Li: Critically commented and revised the manuscript.

Carlos Price-Sanchez: Corrected English and critically commented on the manuscript.

Yuanping Liu, Yanfang Wang, Haoxiang Fu: Coordinated data collection and commented critically on the manuscript.

Nathan Congdon: Guided the study generally and revised the manuscript.

Competing interests: The authors do not have any conflict of interest regarding the study and publication of this article.

Funding: The study was part of "Diabetes Phase II project" funded by Orbis International (Reference No. N/A), World Diabetes Foundation (No. WDF15-1290), Shaoguan Rural Health Express Project (Reference No. N/A) as well as Zhenjiang District Hospital (Reference No. N/A). Prof Nathan Congdon received Wellcome Trust Funding.

Data sharing statement:

The datasets generated and/or analysed during the current study are available from the first and corresponding authors upon reasonable request.

Ethics Approval:

Ethical approval for this study was obtained from Zhongshan Ophthalmic Center, Sun Yat-sen University (Approval Number: 2019KYPJ067).

For peer review only

Acknowledgement:

The authors acknowledge the doctors and nurses from all the ten township health units involved to liaise with the local patients, screening site coordination. Financial support from Shaoguan Rural Health Express program was crucial to the success of the DRS in Qujiang. The trained screening team including Ms Qiong Wan as the team leader from Shaoguan Railway Hospital (Zhenjiang District Hospital) was greatly appreciated.

For peer review only

References:

1. Cho NH, Shaw JE, Karuranga S, et al. IDF Diabetes Atlas: Global estimates of diabetes prevalence for 2017 and projections for 2045. *Diabetes Res Clin Pract* 2018; **138**: 271-81.

2. Ogurtsova K, da Rocha Fernandes JD, Huang Y, et al. IDF Diabetes Atlas: Global estimates for the prevalence of diabetes for 2015 and 2040. *Diabetes Res Clin Pract* 2017; **128**: 40-50.

3. Yang SH, Dou KF, Song WJ. Prevalence of diabetes among men and women in China. *N Engl J Med* 2010; **362**(25): 2425-6; author reply 6.

4. Bragg F, Holmes MV, Iona A, et al. Association Between Diabetes and Cause-Specific Mortality in Rural and Urban Areas of China. *JAMA* 2017; **317**(3): 280-9.

5. Xu Y, Wang L, He J, et al. Prevalence and control of diabetes in Chinese adults. *JAMA* 2013; **310**(9): 948-59.

6. Stratton IM, Kohner EM, Aldington SJ, et al. UKPDS 50: risk factors for incidence and progression of retinopathy in Type II diabetes over 6 years from diagnosis. *Diabetologia* 2001; **44**(2): 156-63.

7. Liu Y, Song Y, Tao L, et al. Prevalence of diabetic retinopathy among 13473 patients with diabetes mellitus in China: a cross-sectional epidemiological survey in six provinces. *BMJ Open* 2017; **7**(1): e013199.

8. Bailey CC, Sparrow JM, Grey RH, Cheng H. The National Diabetic Retinopathy Laser Treatment Audit. II. Proliferative retinopathy. *Eye (Lond)* 1998; **12 (Pt 1)**: 77-84.

9. Kohner EM, Stratton IM, Aldington SJ, Holman RR, Matthews DR, Group UKPDS. Relationship between the severity of retinopathy and progression to photocoagulation in patients with Type 2 diabetes mellitus in the UKPDS (UKPDS 52). *Diabet Med* 2001; **18**(3): 178-84.

10. Mukamel DB, Bresnick GH, Wang Q, Dickey CF. Barriers to compliance with screening guidelines for diabetic retinopathy. *Ophthalmic Epidemiol* 1999; **6**(1): 61-72.

11. Lee PP, Feldman ZW, Ostermann J, Brown DS, Sloan FA. Longitudinal rates of annual eye examinations of persons with diabetes and chronic eye diseases. *Ophthalmology* 2003; **110**(10): 1952-9.

12. Lee SJ, Sicari C, Harper CA, et al. Examination compliance and screening for diabetic retinopathy: a 2-year follow-up study. *Clin Exp Ophthalmol* 2000; **28**(3): 149-52.

13. Schoenfeld ER, Greene JM, Wu SY, Leske MC. Patterns of adherence to diabetes vision care guidelines: baseline findings from the Diabetic Retinopathy Awareness Program. *Ophthalmology* 2001; **108**(3): 563-71.

14. Sloan FA, Brown DS, Carlisle ES, Picone GA, Lee PP. Monitoring visual status: why patients do or do not comply with practice guidelines. *Health Serv Res* 2004; **39**(5): 1429-48.

15. Agarwal S, Mahajan S, Rani PK, et al. How high is the non-response rate of patients referred for eye examination from diabetic screening camps? *Ophthalmic Epidemiol* 2005; **12**(6): 393-4.

16. Lewis K, Patel D, Yorston D, Charteris D. A qualitative study in the United Kingdom of factors influencing attendance by patients with diabetes at ophthalmic outpatient clinics. *Ophthalmic Epidemiol* 2007; **14**(6): 375-80.

17. Thompson AC, Thompson MO, Young DL, et al. Barriers to Follow-Up and Strategies to Improve Adherence to Appointments for Care of Chronic Eye Diseases. *Invest Ophthalmol Vis Sci* 2015; **56**(8): 4324-31.
18. He M, Chan V, Baruwa E, Gilbert D, Frick KD, Congdon N. Willingness to pay for cataract surgery in rural Southern China. *Ophthalmology* 2007; **114**(3): 411-6.
19. Harris M. The NHS Diabetic Eye Screening Programme: New Common Pathway *https://wwwrcophthacuk/wp-content/uploads/2014/08/Focus-Winter-2012pdf* **Accessed March 8th 2022**.
20. Ko F, Frick KD, Tzu J, He M, Congdon N. Willingness to pay for potential enhancements to a low-cost cataract surgical package in rural southern China. *Acta Ophthalmol* 2012; **90**(1): e54-60.
21. Roberts R, Speight L, Lee J, et al. Retinal screening of patients with cystic fibrosis-related diabetes in Wales -- a real eye opener. *J Cyst Fibros* 2015; **14**(2): 282-4.
22. Nagi DK, Gosden C, Walton C, et al. A national survey of the current state of screening services for diabetic retinopathy: ABCD-diabetes UK survey of specialist diabetes services 2006. *Diabet Med* 2009; **26**(12): 1301-5.
23. James M, Turner DA, Broadbent DM, Vora J, Harding SP. Cost effectiveness analysis of screening for sight threatening diabetic eye disease. *BMJ* 2000; **320**(7250): 1627-31.
24. Rathi S, Tsui E, Mehta N, Zahid S, Schuman JS. The Current State of Teleophthalmology in the United States. *Ophthalmology* 2017; **124**(12): 1729-34.
25. Lee SJ, Livingston PM, Harper CA, McCarty CA, Taylor HR, Keeffe JE. Compliance with recommendations from a screening programme for diabetic retinopathy. *Aust N Z J Ophthalmol* 1999; **27**(3-4): 187-9.
26. Lee SJ, McCarty CA, Sicari C, et al. Recruitment methods for community-based screening for diabetic retinopathy. *Ophthalmic Epidemiol* 2000; **7**(3): 209-18.
27. Ting DS, Cheung GC, Wong TY. Diabetic retinopathy: global prevalence, major risk factors, screening practices and public health challenges: a review. *Clin Exp Ophthalmol* 2016; **44**(4): 260-77.
28. Sharma M CA, Pava R, Sharm R, Pratibha G. An integrated, mobile service for diabetic retinopathy in rural India. *Community eye Health/International Centre for Eye Health* 2011; **24**: 17-8.
29. Lin KD, Hsu CC, Ou HY, Wang CY, Chin MC, Shin SJ. Diabetes-related kidney, eye, and foot disease in Taiwan: An analysis of nationwide data from 2005 to 2014. *J Formos Med Assoc* 2019; **118 Suppl 2**: S103-S10.
30. Tung TH, Shih HC, Chen SJ, Chou P, Liu CM, Liu JH. Economic evaluation of screening for diabetic retinopathy among Chinese type 2 diabetics: a community-based study in Kinmen, Taiwan. *J Epidemiol* 2008; **18**(5): 225-33.
31. Klose T. The contingent valuation method in health care. *Health Policy* 1999; **47**(2): 97-123.

For peer review only

STROBE Statement—Checklist of items that should be included in reports of ***cross-sectional studies***

	Item No	Recommendation	Page No
**Title and abstract**	1	(*a*) Indicate the study's design with a commonly used term in the title or the abstract	2
		(*b*) Provide in the abstract an informative and balanced summary of what was done and what was found	2
**Introduction**			
Background/rationale	2	Explain the scientific background and rationale for the investigation being reported	4,5
Objectives	3	State specific objectives, including any prespecified hypotheses	5
**Methods**			
Study design	4	Present key elements of study design early in the paper	5
Setting	5	Describe the setting, locations, and relevant dates, including periods of recruitment, exposure, follow-up, and data collection	5,6,7,8
Participants	6	(*a*) Give the eligibility criteria, and the sources and methods of selection of participants	6
Variables	7	Clearly define all outcomes, exposures, predictors, potential confounders, and effect modifiers. Give diagnostic criteria, if applicable	7,8
Data sources/ measurement	8*	For each variable of interest, give sources of data and details of methods of assessment (measurement). Describe comparability of assessment methods if there is more than one group	*7,8*
Bias	9	Describe any efforts to address potential sources of bias	7,8
Study size	10	Explain how the study size was arrived at	7
Quantitative variables	11	Explain how quantitative variables were handled in the analyses. If applicable, describe which groupings were chosen and why	8,9
Statistical methods	12	(*a*) Describe all statistical methods, including those used to control for confounding	9
		(*b*) Describe any methods used to examine subgroups and interactions	7,8
		(*c*) Explain how missing data were addressed	NiL
		(*d*) If applicable, describe analytical methods taking account of sampling strategy	7
		(*e*) Describe any sensitivity analyses	NiL
**Results**			
Participants	13*	(a) Report numbers of individuals at each stage of study—eg numbers potentially eligible, examined for eligibility, confirmed eligible, included in the study, completing follow-up, and analysed	7
		(b) Give reasons for non-participation at each stage	7
		(c) Consider use of a flow diagram	NiL
Descriptive data	14*	(a) Give characteristics of study participants (eg demographic, clinical, social) and information on exposures and potential confounders	7
		(b) Indicate number of participants with missing data for each variable of interest	7
Outcome data	15*	Report numbers of outcome events or summary measures	9,10
Main results	16	(*a*) Give unadjusted estimates and, if applicable, confounder-adjusted estimates and their precision (eg, 95% confidence interval). Make clear which confounders were adjusted for and why they were included	10

For peer review only

		(*b*) Report category boundaries when continuous variables were categorized	10
		(*c*) If relevant, consider translating estimates of relative risk into absolute risk for a meaningful time period	
Other analyses	17	Report other analyses done—eg analyses of subgroups and interactions, and sensitivity analyses	10
**Discussion**			
Key results	18	Summarise key results with reference to study objectives	10
Limitations	19	Discuss limitations of the study, taking into account sources of potential bias or imprecision. Discuss both direction and magnitude of any potential bias	13,14
Interpretation	20	Give a cautious overall interpretation of results considering objectives, limitations, multiplicity of analyses, results from similar studies, and other relevant evidence	12,13
Generalisability	21	Discuss the generalisability (external validity) of the study results	14
**Other information**			
Funding	22	Give the source of funding and the role of the funders for the present study and, if applicable, for the original study on which the present article is based	15

*Give information separately for exposed and unexposed groups.

Note: An Explanation and Elaboration article discusses each checklist item and gives methodological background and published examples of transparent reporting. The STROBE checklist is best used in conjunction with this article (freely available on the Web sites of PLoS Medicine at http://www.plosmedicine.org/, Annals of Internal Medicine at http://www.annals.org/, and Epidemiology at http://www.epidem.com/). Information on the STROBE Initiative is available at www.strobe-statement.org.

For peer review only

For peer review only - http://bmjopen.bmj.com/site/about/guidelines.xhtml

BMJ Open

Willingness to Pay for Diabetic Retinopathy Screening in Qujiang District, Rural Southern China: A Cross-sectional Study

Journal:	*BMJ Open*
Manuscript ID	bmjopen-2022-065792.R1
Article Type:	Original research
Date Submitted by the Author:	24-Feb-2023
Complete List of Authors:	Xiao, Baixiang; Affiliated Eye Hospital of Nanchang University; Queen's University Belfast,<br>Gu, Xuejun; Affiliated Eye Hospital of Nanchang University<br>Jin, Ling; Sun Yat-Sen University Zhongshan Ophthalmic Center<br>Chan, Ving ; Queen's University Belfast, Center for Public Health<br>Li, Yanping; Nanchang University affiliated Eye Hospital<br>Price-Sanchez, Carlos; Queen’s University Belfast<br>Liu, Yuanping; Sun Yat-Sen University<br>Wang, Yanfang; Zhongshan Ophthalmic Centre, Sun Yat-sen University<br>Fu, Haoxiang; Zhenjiang District Hospital<br>Li, Dongfeng; Queen’s University Belfast<br>Congdon, Nathan; Queen’s University Belfast; Sun Yat-Sen University
**Primary Subject Heading**:	Health economics
Secondary Subject Heading:	Epidemiology, Health services research, Ophthalmology, Public health
Keywords:	Diabetic retinopathy < DIABETES & ENDOCRINOLOGY, EPIDEMIOLOGY, HEALTH ECONOMICS, Health economics < HEALTH SERVICES ADMINISTRATION & MANAGEMENT

SCHOLARONE™
Manuscripts

BMJ

I, the Submitting Author has the right to grant and does grant on behalf of all authors of the Work (as defined in the below author licence), an exclusive licence and/or a non-exclusive licence for contributions from authors who are: i) UK Crown employees; ii) where BMJ has agreed a CC-BY licence shall apply, and/or iii) in accordance with the terms applicable for US Federal Government officers or employees acting as part of their official duties; on a worldwide, perpetual, irrevocable, royalty-free basis to BMJ Publishing Group Ltd ("BMJ") its licensees and where the relevant Journal is co-owned by BMJ to the co-owners of the Journal, to publish the Work in this journal and any other BMJ products and to exploit all rights, as set out in our licence.

The Submitting Author accepts and understands that any supply made under these terms is made by BMJ to the Submitting Author unless you are acting as an employee on behalf of your employer or a postgraduate student of an affiliated institution which is paying any applicable article publishing charge ("APC") for Open Access articles. Where the Submitting Author wishes to make the Work available on an Open Access basis (and intends to pay the relevant APC), the terms of reuse of such Open Access shall be governed by a Creative Commons licence – details of these licences and which Creative Commons licence will apply to this Work are set out in our licence referred to above.

Other than as permitted in any relevant BMJ Author's Self Archiving Policies, I confirm this Work has not been accepted for publication elsewhere, is not being considered for publication elsewhere and does not duplicate material already published. I confirm all authors consent to publication of this Work and authorise the granting of this licence.

For peer review only

Willingness to Pay for Diabetic Retinopathy Screening in Qujiang District, Rural Southern China: A Cross-sectional Study

Baixiang Xiao[1, 2], Xuejun Gu[1], Ling Jin[3], Ving Fai Chan[2,4], Yanping Li[1], Carlos Price-Sanchez[2], Yuanping Liu[3], Yanfang Wang[3], Haoxiang Fu[5], Dongfeng Li[2], Nathan Congdon[2,3,6]

1. Affiliated Eye Hospital of Nanchang University, Nanchang City, China
2. Centre for Public Health, Queen's University Belfast, Belfast, United Kingdom
3. The State Key Laboratory of Ophthalmology, Zhongshan Ophthalmic Center, Sun Yat-sen University, Guangzhou City, China
4. College of Health Sciences, University of KwaZulu Natal. Durban. South Africa
5. Zhenjiang District Hospital, Shaoguan City, Guangdong Province, China
6. Orbis International, NY, United States

Address for correspondence: Prof Nathan Congdon, Centre for Public Health, Royal Victoria Hospital, Queen's University Belfast, 274 Grosvenor Rd, Belfast UK BT12 6BA, and the State Key Laboratory of Ophthalmology, Zhongshan Ophthalmic Center, Sun Yat-sen University, 54 S. Xianlie Road, Guangzhou, People's Republic of China
Email: ncongdon1@gmail.com, Tel: +44 7748751393 (UK) and +86 020 87682342 (P.R.C.)

Keywords: willingness to pay, diabetic retinopathy, screening, rural, China

Word count: 3458

Running head: Willingness to pay for diabetic retinopathy screening
Number of tables: 3
Number of figures: 0

This submission has not been previously published and is not simultaneously being considered by any other publication.

The study was funded by the World Diabetes Foundation, Orbis International, Wellcome Trust Funding and Zhenjiang District Hospital.
Grant number: N/A.

For peer review only

Abstract:

Objective: To determine willingness to pay for a diabetic retinopathy screening (DRS), and its determinants, among people with diabetes mellitus (DM) in Qujiang District of Shaoguan City, rural South China.

Design: This cross-sectional study was conducted through a large scale screening program in 2019. We randomly selected 575 (21.5%) among 2,677 people over 18 years old with known diabetes who attended the screening. Participants elected to pay nothing or RMB10 – 120 (USD 1.6 – 18.8), in RMB 10 intervals, displayed on printed cards. One trained interviewer collected all the data.

Setting: Ten primary health centres in rural Guangdong, southern China.

Participants: Five hundred and fourty-five from the 575 randomly selected people (94.8%) agreed to participate to the study.

Primary and secondary outcome measures: Proportion of participants willing to pay anything for screening, the mean amount willing to pay, and determinants of these figures.

Results: Among 545 participants (mean age 64.6 years [SD±10.4], 40.7% men), 327 (60.0%) were willing to pay something for screening, of whom 273 (83.5%) would pay RMB10 – 30 (USD1.6 – 4.7). People living in rural areas and those from lower-income families were more likely to be willing to pay anything, while men, urban residents, and those covered by employer-linked insurance were willing to pay larger sums ($p<0.05$ for all).

Conclusions and relevance: Nearly two-thirds of participants were willing to pay for screening in this event organized at the primary level in rural China. This offers the potential that such activities can be sustained and scaled up through user fees.

For peer review only

Strengths and limitations of this study:

- Strengths of this study include:
  - Its having been nested in a large-scale, real world screening program in rural China, an area where little is known about willingness to pay for diabetic eye screening services.
  - The use of payment cards of differing amounts, presented simultaneously to participants for selection. This avoided starting point bias in bidding for payment.
  - The upper and lower limits of amounts the study offered for participants' selection were based on the actual cost of retinal detection in hospital and in the community.
- The main weakness is that study was conducted at a single location, and can only be applied to other settings with caution.

For peer review only

Introduction:

The global number of people living with diabetes mellitus (PwDM) has increased steadily from 108 million in 1980 to 425 million in 2017[1]. It is expected to increase to 693 million cases by 2045[2]. The increasing burden of PwDM in China has followed international trends, with the prevalence of adult PwDM increasing from approximately 2% in the 1980s to over 10% in 2017[3-5]. This dramatic rise in PwDM is due to increases in life expectancy, wider adoption of western diets, sedentary lifestyles and expanding urbanization[5].

While diabetes mellitus (DM) itself promises to grow as a health concern, complications associated with its progression pose additional risks to health. DM can lead to ocular complications requiring further ophthalmological care, straining the Chinese public health system. Of these ocular conditions, Diabetic Retinopathy (DR) is the most common and serious, potentially resulting in irreversible blindness. After 20 years, 90% of persons with Type 1 diabetes and 60% with Type 2 will develop some form of retinopathy. A third of these cases will require treatment to prevent irreversible blindness[6]. Given current numbers of PwDM and the prevalence of DR, the need for laser treatments is estimated to be over 8 million in China at present[5,7].

Interventions at the level of primary care are effective at both reducing the severity and slowing the progression of DR. Methods such as adopting a healthy lifestyle to control blood glucose, adhering to prescribed medicines, undergoing regular retinal examinations, and receiving timely treatment for retinopathy with laser photocoagulation, intravitreal injections, or vitrectomy for more serious cases, have together been proven to reduce blindness from DR by

For peer review only

over 90%[8,9]. In particular, establishing robust diabetic retinopathy screening (DRS) protocols at the level of primary care is a key step in preventing the progression of DR towards visual impairment (VI) or blindness (BL).

Despite the efficacy of DRS in preventing vision impairment, increasing the uptake of DRS has been a significant challenge for many health systems[10,11]. Common barriers to uptake include: a lack of perceived need, patient fear of positive diagnosis, high cost, lack of access, and low quality of service[10]. Additionally, DR is often asymptomatic before the disease affects the macula or causes a vitreous haemorrhage and begins to impair visual acuity. Therefore, many unscreened patients are unaware they have the condition before it becomes severe[12-17]. As DR becomes more prevalent, integrating DRS at all levels of service has never been more important, but its benefits may not be obvious to asymptomatic patients.

As part of a pilot program to develop and enhance primary and secondary healthcare models for DRS, Orbis International and the World Diabetes Foundation (WDF) have supported eye care services for PwDM at 6 county hospitals in Guangdong Province since 2017. The current willingness to pay (WTP) study was conducted as part of the DRS program in Qujiang District of Shaoguan City, northern Guangdong Province, in the second half of 2019. Though prior studies in the area have examined WTP for cataract surgery[18], at present, no such studies on DRS have been completed in China. This study aims to fill this gap by exploring WTP for DRS services in rural North Guangdong Province. Despite the current lack of targeted funding for DRS in most areas of China, charging for this crucial service might be sufficient to sustain the service if beneficiaries are willing to pay enough. The findings in this report will provide context for future decisions regarding providing care for PwDM and establishing community

For peer review only

DRS services in rural China.

Methods

Ethical approval for the DRS study in Qujiang, south rural China, was obtained from Zhongshan Ophthalmic Center, Sun Yat-sen University (ZOC) (Approval Number: 2019KYPJ067). Written consent was obtained from each participant. PwDM found with sight-threatening DR (R2 or above in the UKNHS DRS system[19]), operable cataracts, or other suspected eye diseases were referred to secondary-level hospitals for further examination or treatment. The study fulfilled the tenets of the Declaration of Helsinki.

Setting and study participants:

Qujiang is a secondary-level district in North Guangdong province of China, home to 320,000 people in 2018 (a population density of 192 people per km^2), with a 2018 per capita gross domestic product (GDP) of USD7,160.32. Qujiang's GDP is significantly less than the national average per capita GDP of USD10,253.97 in the same year. These figures are typical for other poor rural areas in Southern China.

Shaoguan City Railway Hospital and Qujiang County Hospital are secondary-level hospitals in Zhenjiang and Qujiang Districts respectively. Two doctors from the eye department of Shaoguan City Railway hospital were trained in detecting and treating DR with fundus laser and intra-vitreous injection. Trainers for DR screening and managers were also trained in the program to arrange periodic, small-scale DRS in rural township health facilities and urban

For peer review only

community health centers since 2017. Basic examinations for anterior segment eye disease were provided at the outpatient department in Qujiang Hospital. Patients suspected of having posterior segment diseases, including DR, would have to travel to the neighbouring district hospital in Zhenjiang or a facility in the city centre.

As in many other places in China, PwDM are encouraged to register with the primary health system in Qujiang District for a free annual health check, monitoring of complications, and health education. Village doctors (VDs) provide free fasting blood glucose checks for people aged over 35 years or anyone wishing to test their glucose levels. People suspected of having DM are referred for further examination at secondary-level hospitals. A total of 3,646 PwDM registered across 10 primary health centres in Qujiang District were informed of these local health bureau-supported DRS services, at which point they were able to schedule appointments with trained VDs.

Study team members at the screening sites included an ophthalmologist, two nurses, one technician, and one interviewer, all trained for the study. PwDM came to the township health units for the screening at a pre-scheduled time.

Of the 3,646 PwDM informed about the screening, 2,677 (73.4%) from across the 10 towns in Qujiang attended. This was the first formally arranged, large-scale DRS conducted in the district. We randomly approached 575 PwDM (21.5%) attending the DRS by generating a random number for the selection of a first participant and then approaching every sixth participant from that point on who arrived for screening. In total, 545 (94.8%) of those approached agreed to participate in the WTP study and were recruited.

For peer review only

Patient and public involvement statement:

All participants were informed of the scheduled DRS 1-2 months before screening started, through the trained VDs in the study area and posters circulated in the villages. They were informed of the WTP study by the interviewer at the screening site. Participants were not directly involved in designing the study, although draft questionnaires were tested with some PwDM.

Data collection:

Participants were welcomed to the scheduled annual DR screening by their local public health doctor at each screening site. The local physician would then personally inform each participant when their screening reports were available via mobile phone, text, or WeChat message within one week. Participants were then supplied with DR educational bulletins and posters, which provided information on DR and highlighted the importance of receiving annual fundus imaging for the early detection of ocular diseases.

Participants' basic information and disease history were collected and double-checked via the Chronic Disease Recording System. Retrieved data included participant name, age, sex, site of residence (rural or urban), education, profession, insurance, self-assessment of visual acuity, economic situation including the proportion of family income spent on food, and history of diseases including DM and hypertension. Venous blood was collected from each participant to monitor HbA1c. Presenting visual acuity (PVA) was tested monocularly in each eye at 6 meters with a retro-illuminated Snellen visual chart before the ophthalmologist carried out a slit lamp examination. Intraocular pressure (IOP) was measured in each eye using tono-pen

For peer review only

before pupils were dilated with compound tropicamide to enable the capture of fundus images with a Canon CR-2 by a trained nurse. Patients with high IOP or shallow anterior chambers detected under slit lamp examination were not dilated; instead, images were taken through pupils which had been dilated scotopically after around 3 to 5 minutes in a darkened room.

Diabetic complications were defined as any of the following symptoms: ulcers on hands or feet, cardiovascular disease, diabetic nephropathy, diabetic eye disease, ketoacidosis or other serious metabolic complications. Participants could self-assess their physical health and visual acuity on a scale of 1-5, corresponding to: bad (1), not good (2), alright (3), good (4) and excellent (5). Regarding their economic situation, respondents were given two choices related to the proportion of monthly family income spent on food: less than 30%, equal to or over 30%.

Questionnaires regarding WTP for the DR screening were administered post screening in a quiet, separate room. Each participant was given the opportunity to ask any questions they might have on DM, DR, screening, or the report, and received informed responses from the trained interviewer. Comments on the screening were also invited. The participant was then told that the screening required appropriate human resources, equipment, and preparation, so there was some cost. The direct cost (travel or transport cost, daily allowance for screening staff) of one person screened was approximately USD4.7. At the time of the study, there was no budget in the primary health care system for the DRS. According to the provincial pricing regulations, fundus imaging charges were USD 18.8 for both eyes at secondary level hospitals. Ultimately these costs fall on the patient, as expenses associated with imaging and registration are not currently reimbursed by insurance.

For peer review only

Each participant was informed that their choice of willingness to pay category would not affect their ability to receive primary health care, medical services in hospitals, nor current or any future DR screening. Participants were first asked whether they would like to pay something, starting at 10 yuan (USD 1.6), for the screening. Reasons were elicited for those answering "no". When a participant expressed willingness to pay any amount, they were presented with cards marked from 10 yuan (USD 1.6) to 120 yuan (USD 18.8) at intervals of 10 yuan (USD 1.6) (12 cards in total). To avoid bargaining bias, we presented participants with cards valued at RMB10, 20, 30, 40, 50, 60, 70, 80, 90, 100, 110 and 120 simultaneously for their selection. A trained interviewer collected all data for WTP to maintain the consistency and quality of the interview, although general data were collected by three trained staff.

Statistical analysis:

Participants' characteristics were presented as mean (standardised deviation, SD) for continuous data with normal distribution or median (inter quartile range, IQR) for data without normal distribution, and frequency (%) for categorical variables. Shapiro–Wilk normality tests and histograms were applied to check the normality of continuous data. Participants' age was categorized by intervals of 10 years. Duration of diabetes was categorized by intervals of 5 years. The cut-offs of 6/60, 6/18 and 6/12 were used to describe visual acuity in the better-seeing eye.

Logistic regression analysis for WTP and linear regression analysis for the different amounts participants would pay were employed to examine the associations between potential factors

For peer review only

and outcomes. Univariable and multivariable regression models were fitted. Age, sex and variables with P<0.20 in the univariable analysis were included in the multivariable regression model. All statistical analyses were performed using a commercially available software package (Stata 16, Stata Corp, College Station TX, USA).

The reasons given by the participants who would not pay anything for the DRS at community level were listed in text including all the points mentioned.

Results:

Among 545 participants, the mean age was 64.4±10.4 years (range 35-92 years), and 323 [59.3%] were women. (Table 1) A total of 345 (63.3%) participants lived in urban settings. Almost half (44.6%) of participants had received a primary school education and 54 (9.9%) attended high school or above. All participants had either standard residential medical insurance (n=372, 68.3%) or employment-linked insurance (n=173, 31.7%).

For peer review only

Table 1. Demographic characteristic of participants in the willingness to pay study, Qujiang, China

Characteristic	Mean (SD)/n (%)
Total	545
**Age (year), mean (SD)**	64.6 (10.4)
<50	48 (8.8)
50-59	131(24.0)
60-69	198(36.3)
70-79	137(25.1)
80+	31(5.7)
**Gender, n (%)**	
Male	222 (40.7)
Female	323 (59.3)
**Duration of DM (year), n (%)**	
<5	279 (51.2)
5-10	130 (23.9)
10-15	73 (13.4)
>15	63 (11.6)
**Diabetic complications present, n (%)**	
Yes	434 (79.6)
No	111 (20.4)
**Residence, n (%)**	
Urban	345 (63.3)
Rural	200 (36.7)
**Education, n (%)**	
Never received formal education	81 (14.9)
Primary school	243 (44.6)
Junior high school	167 (30.6)
High school or above	54 (9.9)
**Presenting visual acuity in better-seeing eye, n (%)**	
>=6/12	435 (79.8)
>/=6/18 to <6/12	72 (13.2)
>/=6/60 to <6/18	32 (5.9)
<6/60	6 (1.1)
**Medical insurance, n (%)**	
Residential medical insurance	372 (68.3)
Employment-linked insurance	173 (31.7)
**Self-assessment of current health situation, n (%)**	
Bad	11 (2.02)
Not good	107 (19.6)
Good	261 (47.9)
Very good	153 (28.1)
Excellent	13 (2.39)
**Self-assessment of current visual acuity, n (%)**	
Bad	12 (2.20)
Not good	101 (18.5)
Good	349 (64.0)
Very good	74 (13.6)
Excellent	9 (1.65)
**Economic situation, n (%)**	
<30% of monthly family income spent on food	108 (19.8)
⩾30% of monthly family income spent on food	437 (80.2)

SD: Standard deviation

For peer review only

Approximately half (51.2%) of participants had been diagnosed with DM within the last 5 years and only 63 (11.6%) had been diagnosed over 15 years prior (Table 1). Most participants (n=435, 79.8%) had presenting VA >= 6/12 in the better-seeing eye. Most participants (n=434, 79.6%) did not report any diabetic complications. Nearly 80% of participants reported being in good, very good, or excellent health at the time of the screening, and a similar proportion described their vision as falling in the same three categories. Regarding their economic situation, most participants (80.2%) reported their households spent 30% or above of their monthly family income on food, while 19.8% spent less than 30%. (Table 1)

Overall, 327 (60.0%) participants expressed a willingness to pay something for the DRS at the primary level, of whom 273 (83.5%) would pay RMB10 – 30 (USD 1.6 – 4.7), 34 (10.4%) would pay RMB 40 – 50 (USD 6.3 – 7.8), and 15 (4.6%) would pay RMB 80 -120 (USD 12.5 – 18.8). In logistic regression models, only rural residence and those paying >= 30% of income for food (both more willing) were associated with willingness to pay something for DRS (Table 2). Both variables were significant in the univariate and multivariate models.

Among 327 PwDM who were willing to pay something for screening, in univariate linear regression models, men, urban residents, and those with higher educational levels and covered by employment-linked insurance were willing to pay more (Table 3). Conversely, participants with worse self-rated health and vision were willing to pay less. Only the associations with male sex, urban residence and employment-linked health insurance remained significant in the multivariate models. The level of HbA1C were not found having significant association with willingness to pay for DRS.

For peer review only

Table 2. Logistic Analysis of Willingness to Pay for Diabetic Retinopathy Screening, Qujiang, China (n=545)

Characteristic	Univariable regression		Multivariable regression* (n=545)	
	OR (95% CI)	P	OR (95% CI)	P
Age (per year)	0.99 (0.97, 1.01)	0.217	1.00 (0.98, 1.02)	0.690
Female sex	0.83 (0.59, 1.18)	0.302	0.98 (0.66, 1.44)	0.903
Duration of DM (year)				
<5	Reference		Reference	
5-10	0.67 (0.44, 1.03)	0.066	0.71 (0.46, 1.11)	0.138
10-15	0.64 (0.38, 1.08)	0.094	0.75 (0.43, 1.30)	0.306
>15	0.85 (0.48, 1.49)	0.568	1.18 (0.64, 2.19)	0.591
Diabetic Complications	0.89 (0.58, 1.37)	0.602		
Urban residence	0.46 (0.31, 0.66)	<0.001	0.44 (0.28, 0.69)	<0.001
Education				
Never received formal education	Reference		Reference	
Primary school	1.07 (0.64, 1.78)	0.795	1.31 (0.75, 2.28	0.350
Junior high school	1.16 (0.68, 1.99)	0.579	1.39 (0.74, 2.63	0.310
High school or above	1.81 (0.87, 3.75)	0.113	2.07 (0.90, 4.78	0.087
Presenting visual acuity in better-seeing eye				
>/=6/12	Reference			
>/=6/18 to <6/12	0.93 (0.51, 1.71)	0.823		
>/=6/60 to <6/18	1.03 (0.58, 1.82)	0.918		
<6/60	1.33 (0.24, 7.36)	0.741		
Standard residential health insurance	1.41 (0.98, 2.03)	0.066	0.94 (0.59, 1.51)	0.804
Self-assessment of health				
Bad	Reference			
Not good	0.96 (0.26, 3.47)	0.947		
Good	0.70 (0.20, 2.46)	0.582		
Very good	1.02 (0.29, 3.63)	0.978		
Excellent	3.14 (0.45, 22.0)	0.248		
Self-assessment of visual acuity				
Bad	Reference		Reference	
Not good	1.00 (0.30, 3.38)	0.996	1.09 (0.30, 3.92)	0.894
Good	0.96 (0.30, 3.08)	0.944	0.74 (0.21, 2.55)	0.629

For peer review only

Very good	1.80 (0.51, 6.32)	0.357	1.13 (0.29, 4.37)	0.854
Excellent	5.71 (0.53, 61.4)	0.150	4.03 (0.34, 47.5)	0.268
Economic situation				
<30% monthly income on food	Reference		Reference	
⩾30% on food	1.66 (1.09, 2.54)	0.018	1.70 (1.04, 2.77)	0.035

OR: Odds ratio, CI: Confidence interval

* Age, sex and variables with P<0.20 in the univariable regression analysis were included in the multivariable regression model.

For peer review only

Table 3. Linear Regression of Subjects Willing to Pay Something for Diabetic Retinopathy Screening, Qujiang, China (N=327) *

Characteristic	Univariable regression		Multivariable regression† (n=327)	
	□ (95% CI)	P	□ (95% CI)	P
Age (per year)	-0.0003 (-0.003, 0.002)	0.847	-0.002 (-0.004, 0.001)	0.281
Female sex	-0.10 (-0.16, -0.05)	<0.001	-0.07 (-0.13, -0.02)	0.013
Duration of DM (year)				
<5	Reference			
5-10	0.02 (-0.05, 0.09)	0.628		
10-15	0.006 (-0.08, 0.10)	0.890		
>15	0.05 (-0.04, 0.14)	0.283		
Diabetic Complications Present	0.04 (-0.03, 0.11)	0.300		
Urban residence	0.16 (0.10, 0.21)	<0.001	0.14 (0.07, 0.20)	<0.001
Education				
Never received formal education	Reference		Reference	
Primary school	0.15 (0.06, 0.23)	0.001	0.06 (-0.02, 0.14)	0.162
Junior high school	0.13 (0.04, 0.22)	0.005	0.0001 (-0.10, 0.10)	0.998
High school or above	0.22 (0.11, 0.33)	<0.001	0.09 (-0.03, 0.20)	0.149
Presenting visual acuity in better-seeing eye				
>/=6/12	Reference		Reference	
>/=6/18 to <6/12	-0.04 (-0.14, 0.07)	0.496	-0.005 (-0.10, 0.09)	0.911
>/=6/60 to <6/18	-0.08 (-0.18, 0.01)	0.080	-0.02 (-0.11, 0.07)	0.707
<6/60	0.05 (-0.21, 0.30)	0.718	0.17 (-0.08, 0.42)	0.173
Standard residential health insurance	-0.15 (-0.21, -0.09)	<0.001	-0.08 (-0.15, -0.004)	0.040
Self-assessment of health				
Bad	Reference		Reference	
Not good	-0.27 (-0.47, -0.07)	0.009	-0.13 (-0.47, 0.21)	0.455
Good	-0.22 (-0.42, -0.02)	0.028	-0.07 (-0.43, 0.28)	0.696
Very good	-0.26 (-0.46, -0.06)	0.010	-0.08 (-0.43, 0.28)	0.672
Excellent	-0.22 (-0.47, 0.02)	0.073	-0.32 (-0.73, 0.10)	0.134
Self-assessment of VA				
Bad	Reference		Reference	
Not good	-0.28 (-0.48, -0.08)	0.007	-0.11 (-0.46, 0.24)	0.532
Good	-0.23 (-0.43, -0.04)	0.018	-0.05 (-0.41, 0.31)	0.781
Very good	-0.27 (-0.47, -0.07)	0.008	-0.06 (-0.42, 0.31)	0.756
Excellent	-0.03 (-0.29, 0.23)	0.835	0.33 (-0.10, 0.76)	0.135
Economic situation				
<30% income paid for food	Reference			
⩾30% income paid for food	0.007 (-0.07, 0.08)	0.847		

□: Parameter coefficient estimate, CI: Confidence Interval

* The amount willing to pay was not normally distributed and logarithmic transformation was applied.

† Age, sex and variables with P<0.20 in the univariable regression analysis were included in the multivariable regression model.

Reasons given by participants unwilling to pay anything for the DRS included: should be covered by government or medical insurance; screening report was not received so cannot tell the quality of this service; suspicious whether the money charged would be used properly; suspicious whether the program would be sustainable; would rather go to hospital.

For peer review only

Discussion:

In this study, among 545 randomly selected people with diagnosed diabetes who attended a large scale, primary care diabetic retinopathy screening in rural south China, nearly two-thirds (60%) were willing to pay something for diabetic retinopathy screening. The groups most likely to be willing to pay something were rural-dwellers and economically less-advantaged families. Among participants willing to pay something, those willing to pay more included men, urban-dwellers, and those covered by employment-linked insurance. This study has highlighted a considerable willingness to pay for these services among primary care patients.

The other WTP study for eye health services we could identify in China, conducted in 2006 on cataract surgeries, established that 73% of cataract patients were willing to pay something, and that many were willing to pay more for experienced surgeons and higher-quality intra-ocular lenses[20]. Possible reasons for a lower proportion willing to pay anything for DRS as compared to cataract might include the belief that DRS is a public health service and therefore should be paid for by the government; cataract surgeries are in most cases associated with significant improvements in vision; and coverage rates of medical insurance have increased in China since 2008, potentially reducing willingness to pay out of pocket for healthcare services.

Many countries have experimented with DR screening models to reduce barriers to access and increase service uptake. For example, the United Kingdom and some other European countries have established free community screening for DR[21,22] and found it to be cost-effective[23]. Tele-ophthalmology and general practitioners have been used in the United States, with services either reimbursed by insurance, or paid fully or in part out-of-pocket[24]. General practitioners'

For peer review only

referrals for those detected at DR screenings to retinal specialists for further treatment were shown to be effective in preventing VI from DR in Australia[25,26]. There are also established DR screening services in Asian countries such as Singapore[27], India[27,28] and Taiwan[29].

This study suggests that rural residents and those who are less economically advantaged are willing to pay more for DRS services. In Qujiang District, Shaoguan City, rural residents with DM would otherwise have to travel to the neighboring district hospital or the city centre to seek eye services. Given that there is no public transportation, this trip would take over an hour. Traveling by motorcycle was the most common mode used by rural residents, making an hour-long trip difficult. Additionally, many elderly could only be taken to the city or the neighboring district with the help of a younger person. The fact that this program increased ease of access by bringing DRS services into rural areas may explain why rural residents were more likely to be willing to pay for screening at their township health unit.

Our study found that people covered by employment-linked insurance were more willing to pay higher fees for DRS. This is likely due to their having comparatively better medical insurance and possibly higher incomes. Unfortunately, it was not possible to collect information on income to test this hypothesis in the current study.

In the current study, men and urban residents were willing to pay more for DRS. This could potentially be explained by their being more economically advantaged on average, or their having greater awareness of the importance of DRS. Urban residents may be more apt to understand the need for examinations to prevent diabetic complications and this suggests that future health education interventions should target rural residents, especially rural women.

For peer review only

Another study has reported that more severe DR among persons with type 2 diabetes was associated with lower perceived utility from the patient's preference-based viewpoint[30]. We did not analyse this metric as the number of patients at R3 was not great enough (9 people, 1.7% among the 545 participants). Rather, we compared the self-assessment of visual acuity and WTP, but found no association between WTP and self-assessed health or vision.

Strengths of the current study include its relevance to actual screening activities, having been community-based, and incorporated into a scheduled, large-scale DR screening program supported by the local hospital, health authorities, and primary level health staff. Additionally, to determine the sum participants were willing to pay, we used a direct method with payment cards of differing amounts which were presented simultaneously to participants for selection. This avoided starting point bias in bidding for payment. To avoid range bias[31], we used 10 yuan (USD 1.6) for a small-scale stable range which would not greatly affect the payment value. The minimum value of 10 yuan is equal to the common consultant fee for a hospital outpatient department, and the maximum value of 120 yuan (USD 19.01) is equal to the sum charged for two-eye fundus images taken at secondary level hospitals. The presented value on payment cards was empirical and conceptual, taking account of the actual pricing of ocular medical services as well as the inherent monetary value of 10 yuan (USD 1.6).

Weaknesses must also be acknowledged: The study was conducted at a single point in time and cannot reflect long-term WTP values as the economy is constantly developing. The study also took place in one mostly rural region, so it does not represent other areas in China. This WTP study is hypothetical and assumes that screening is beneficial to the health of participants.

For peer review only

Cost of screening was introduced and reinforced by eliciting payment amounts. We have also assumed that the respondents were willing to accept the screening as a benefit to their health. The amounts which participants reported they were willing to pay and the amounts they would actually pay may not be the same[31].

To our knowledge, this is the first study on WTP for DRS in China. Cost has been highlighted as the major barrier to establishing DRS at the primary level in China, outside of wealthy areas such as Shanghai, where DRS is well established and fully covered by the metropolitan city government. Findings from this WTP study provide evidence that substantial screening costs could potentially be covered for by patients.

While a considerable percentage of interviewed PwDM demonstrated willingness to take on some personal cost for DRS, studies have noted that organised screening programs result in substantial cost savings for national health systems[19,30]. Recognising that some 50% of current national medical expenditures in China are covered by government medical insurance, investment to establish systematic DRS at the primary care level could substantially reduce costs for the national healthcare system, as well as reducing out-of-pocket medical expenditures[19]. Conducting additional studies in urban areas, as well as in other diverse settings, would contribute to a more complete picture of WTP for DRS in China and help guide the establishment of organised DRS programs. To all the stakeholders of this project, sustainability of the current program would take a key role to serve PwDM in the areas.

Conclusions:

Nearly two-thirds of participants were willing to pay for screening in this event organized at

For peer review only

the primary level in rural China, which offers the potential that such activities can be sustained and scaled up through user fees.

For peer review only

Contributorship statement:

Baixiang Xiao: Designed and monitored the whole study, initially drafted and revised the manuscript.

Ling Jin: Analyzed data.

Xuejun Gu, Ving Fai Chan, Yanping Li, Dongfeng Li: Critically commented and revised the manuscript.

Carlos Price-Sanchez: Corrected English and critically commented on the manuscript.

Yuanping Liu, Yanfang Wang, Haoxiang Fu and Yichun Zhong: Coordinated data collection and commented critically on the manuscript.

Nathan Congdon: Guided the study generally and revised the manuscript.

Competing interests: The authors do not have any conflict of interest regarding the study and publication of this article.

Funding: The study was part of "Diabetes Phase II project" funded by Orbis International (Reference No. N/A), World Diabetes Foundation (No. WDF15-1290), Shaoguan Rural Health Express Project (Reference No. N/A) as well as Zhenjiang District Hospital (Reference No. N/A). Prof Nathan Congdon received Wellcome Trust Funding.

Data sharing statement:

The datasets generated and/or analysed during the current study are available from the first and corresponding authors upon reasonable request.

Ethics Approval:

Ethical approval for this study was obtained from Zhongshan Ophthalmic Center, Sun Yat-sen University (Approval Number: 2019KYPJ067).

For peer review only

Acknowledgement:

The authors acknowledge the doctors and nurses from all the ten township health units involved to liaise with the local patients, screening site coordination. Financial support from Shaoguan Rural Health Express program was crucial to the success of the DRS in Qujiang. The trained screening team including Ms Qiong Wan as the team leader from Shaoguan Railway Hospital (Zhenjiang District Hospital) was greatly appreciated.

References:

1. Cho NH, Shaw JE, Karuranga S, et al. IDF Diabetes Atlas: Global estimates of diabetes prevalence for 2017 and projections for 2045. *Diabetes Res Clin Pract* 2018; **138**: 271-81.
2. Ogurtsova K, da Rocha Fernandes JD, Huang Y, et al. IDF Diabetes Atlas: Global estimates for the prevalence of diabetes for 2015 and 2040. *Diabetes Res Clin Pract* 2017; **128**: 40-50.
3. Yang SH, Dou KF, Song WJ. Prevalence of diabetes among men and women in China. *N Engl J Med* 2010; **362**(25): 2425-6; author reply 6.
4. Bragg F, Holmes MV, Iona A, et al. Association Between Diabetes and Cause-Specific Mortality in Rural and Urban Areas of China. *JAMA* 2017; **317**(3): 280-9.
5. Xu Y, Wang L, He J, et al. Prevalence and control of diabetes in Chinese adults. *JAMA* 2013; **310**(9): 948-59.
6. Stratton IM, Kohner EM, Aldington SJ, et al. UKPDS 50: risk factors for incidence and progression of retinopathy in Type II diabetes over 6 years from diagnosis. *Diabetologia* 2001; **44**(2): 156-63.
7. Liu Y, Song Y, Tao L, et al. Prevalence of diabetic retinopathy among 13473 patients with diabetes mellitus in China: a cross-sectional epidemiological survey in six provinces. *BMJ Open* 2017; **7**(1): e013199.
8. Bailey CC, Sparrow JM, Grey RH, Cheng H. The National Diabetic Retinopathy Laser Treatment Audit. II. Proliferative retinopathy. *Eye (Lond)* 1998; **12 (Pt 1)**: 77-84.
9. Kohner EM, Stratton IM, Aldington SJ, Holman RR, Matthews DR, Group UKPDS. Relationship between the severity of retinopathy and progression to photocoagulation in patients with Type 2 diabetes mellitus in the UKPDS (UKPDS 52). *Diabet Med* 2001; **18**(3): 178-84.
10. Mukamel DB, Bresnick GH, Wang Q, Dickey CF. Barriers to compliance with screening guidelines for diabetic retinopathy. *Ophthalmic Epidemiol* 1999; **6**(1): 61-72.
11. Lee PP, Feldman ZW, Ostermann J, Brown DS, Sloan FA. Longitudinal rates of annual eye examinations of persons with diabetes and chronic eye diseases. *Ophthalmology* 2003; **110**(10): 1952-9.
12. Lee SJ, Sicari C, Harper CA, et al. Examination compliance and screening for diabetic retinopathy: a 2-year follow-up study. *Clin Exp Ophthalmol* 2000; **28**(3): 149-52.
13. Schoenfeld ER, Greene JM, Wu SY, Leske MC. Patterns of adherence to

For peer review only

diabetes vision care guidelines: baseline findings from the Diabetic Retinopathy Awareness Program. *Ophthalmology* 2001; **108**(3): 563-71.
14. Sloan FA, Brown DS, Carlisle ES, Picone GA, Lee PP. Monitoring visual status: why patients do or do not comply with practice guidelines. *Health Serv Res* 2004; **39**(5): 1429-48.
15. Agarwal S, Mahajan S, Rani PK, et al. How high is the non-response rate of patients referred for eye examination from diabetic screening camps? *Ophthalmic Epidemiol* 2005; **12**(6): 393-4.
16. Lewis K, Patel D, Yorston D, Charteris D. A qualitative study in the United Kingdom of factors influencing attendance by patients with diabetes at ophthalmic outpatient clinics. *Ophthalmic Epidemiol* 2007; **14**(6): 375-80.
17. Thompson AC, Thompson MO, Young DL, et al. Barriers to Follow-Up and Strategies to Improve Adherence to Appointments for Care of Chronic Eye Diseases. *Invest Ophthalmol Vis Sci* 2015; **56**(8): 4324-31.
18. He M, Chan V, Baruwa E, Gilbert D, Frick KD, Congdon N. Willingness to pay for cataract surgery in rural Southern China. *Ophthalmology* 2007; **114**(3): 411-6.
19. Harris M. The NHS Diabetic Eye Screening Programme: New Common Pathway https://wwwrcophthacuk/wp-content/uploads/2014/08/Focus-Winter-2012pdf **Accessed March 8th 2022**.
20. Ko F, Frick KD, Tzu J, He M, Congdon N. Willingness to pay for potential enhancements to a low-cost cataract surgical package in rural southern China. *Acta Ophthalmol* 2012; **90**(1): e54-60.
21. Roberts R, Speight L, Lee J, et al. Retinal screening of patients with cystic fibrosis-related diabetes in Wales -- a real eye opener. *J Cyst Fibros* 2015; **14**(2): 282-4.
22. Nagi DK, Gosden C, Walton C, et al. A national survey of the current state of screening services for diabetic retinopathy: ABCD-diabetes UK survey of specialist diabetes services 2006. *Diabet Med* 2009; **26**(12): 1301-5.
23. James M, Turner DA, Broadbent DM, Vora J, Harding SP. Cost effectiveness analysis of screening for sight threatening diabetic eye disease. *BMJ* 2000; **320**(7250): 1627-31.
24. Rathi S, Tsui E, Mehta N, Zahid S, Schuman JS. The Current State of Teleophthalmology in the United States. *Ophthalmology* 2017; **124**(12): 1729-34.
25. Lee SJ, Livingston PM, Harper CA, McCarty CA, Taylor HR, Keeffe JE. Compliance with recommendations from a screening programme for diabetic retinopathy. *Aust N Z J Ophthalmol* 1999; **27**(3-4): 187-9.
26. Lee SJ, McCarty CA, Sicari C, et al. Recruitment methods for community-based screening for diabetic retinopathy. *Ophthalmic Epidemiol* 2000; **7**(3): 209-18.
27. Ting DS, Cheung GC, Wong TY. Diabetic retinopathy: global prevalence, major risk factors, screening practices and public health challenges: a review. *Clin Exp Ophthalmol* 2016; **44**(4): 260-77.
28. Sharma M CA, Pava R, Sharm R, Pratibha G. An integrated, mobile service for diabetic retinopathy in rural India. *Community eye Health/International Centre for Eye Health* 2011; **24**: 17-8.
29. Lin KD, Hsu CC, Ou HY, Wang CY, Chin MC, Shin SJ. Diabetes-related kidney, eye, and foot disease in Taiwan: An analysis of nationwide data from 2005 to 2014. *J Formos Med Assoc* 2019; **118 Suppl 2**: S103-S10.
30. Tung TH, Shih HC, Chen SJ, Chou P, Liu CM, Liu JH. Economic evaluation of screening for diabetic retinopathy among Chinese type 2 diabetics: a community-

For peer review only

based study in Kinmen, Taiwan. *J Epidemiol* 2008; **18**(5): 225-33.
31. Klose T. The contingent valuation method in health care. *Health Policy* 1999; **47**(2): 97-123.

For peer review only

STROBE Statement—Checklist of items that should be included in reports of ***cross-sectional studies***

	Item No	Recommendation	Page No
**Title and abstract**	1	(*a*) Indicate the study's design with a commonly used term in the title or the abstract	2
		(*b*) Provide in the abstract an informative and balanced summary of what was done and what was found	2
**Introduction**			
Background/rationale	2	Explain the scientific background and rationale for the investigation being reported	4,5
Objectives	3	State specific objectives, including any prespecified hypotheses	5
**Methods**			
Study design	4	Present key elements of study design early in the paper	5
Setting	5	Describe the setting, locations, and relevant dates, including periods of recruitment, exposure, follow-up, and data collection	5,6,7,8
Participants	6	(*a*) Give the eligibility criteria, and the sources and methods of selection of participants	6
Variables	7	Clearly define all outcomes, exposures, predictors, potential confounders, and effect modifiers. Give diagnostic criteria, if applicable	7,8
Data sources/ measurement	8*	For each variable of interest, give sources of data and details of methods of assessment (measurement). Describe comparability of assessment methods if there is more than one group	*7,8*
Bias	9	Describe any efforts to address potential sources of bias	7,8
Study size	10	Explain how the study size was arrived at	7
Quantitative variables	11	Explain how quantitative variables were handled in the analyses. If applicable, describe which groupings were chosen and why	8,9
Statistical methods	12	(*a*) Describe all statistical methods, including those used to control for confounding	9
		(*b*) Describe any methods used to examine subgroups and interactions	7,8
		(*c*) Explain how missing data were addressed	NiL
		(*d*) If applicable, describe analytical methods taking account of sampling strategy	7
		(*e*) Describe any sensitivity analyses	NiL
**Results**			
Participants	13*	(a) Report numbers of individuals at each stage of study—eg numbers potentially eligible, examined for eligibility, confirmed eligible, included in the study, completing follow-up, and analysed	7
		(b) Give reasons for non-participation at each stage	7
		(c) Consider use of a flow diagram	NiL
Descriptive data	14*	(a) Give characteristics of study participants (eg demographic, clinical, social) and information on exposures and potential confounders	7
		(b) Indicate number of participants with missing data for each variable of interest	7
Outcome data	15*	Report numbers of outcome events or summary measures	9,10
Main results	16	(*a*) Give unadjusted estimates and, if applicable, confounder-adjusted estimates and their precision (eg, 95% confidence interval). Make clear which confounders were adjusted for and why they were included	10

For peer review only

For peer review only - http://bmjopen.bmj.com/site/about/guidelines.xhtml

		(*b*) Report category boundaries when continuous variables were categorized	10
		(*c*) If relevant, consider translating estimates of relative risk into absolute risk for a meaningful time period	
Other analyses	17	Report other analyses done—eg analyses of subgroups and interactions, and sensitivity analyses	10
**Discussion**			
Key results	18	Summarise key results with reference to study objectives	10
Limitations	19	Discuss limitations of the study, taking into account sources of potential bias or imprecision. Discuss both direction and magnitude of any potential bias	13,14
Interpretation	20	Give a cautious overall interpretation of results considering objectives, limitations, multiplicity of analyses, results from similar studies, and other relevant evidence	12,13
Generalisability	21	Discuss the generalisability (external validity) of the study results	14
**Other information**			
Funding	22	Give the source of funding and the role of the funders for the present study and, if applicable, for the original study on which the present article is based	15

*Give information separately for exposed and unexposed groups.

Note: An Explanation and Elaboration article discusses each checklist item and gives methodological background and published examples of transparent reporting. The STROBE checklist is best used in conjunction with this article (freely available on the Web sites of PLoS Medicine at http://www.plosmedicine.org/, Annals of Internal Medicine at http://www.annals.org/, and Epidemiology at http://www.epidem.com/). Information on the STROBE Initiative is available at www.strobe-statement.org.

For peer review only

BMJ Open

Willingness to pay for diabetic retinopathy screening in Qujiang District, rural Guangdong, southern China: a cross-sectional study

Journal:	*BMJ Open*
Manuscript ID	bmjopen-2022-065792.R2
Article Type:	Original research
Date Submitted by the Author:	18-Mar-2023

Complete List of Authors:	Xiao, Baixiang; Affiliated Eye Hospital of Nanchang University; Queen's University Belfast,<br>Gu, Xuejun; Affiliated Eye Hospital of Nanchang University<br>Jin, Ling; Sun Yat-Sen University Zhongshan Ophthalmic Center<br>Chan, Ving ; Queen's University Belfast, Center for Public Health<br>Li, Yanping; Nanchang University affiliated Eye Hospital<br>Price-Sanchez, Carlos; Queen's University Belfast<br>Liu, Yuanping; Sun Yat-Sen University<br>Wang, Yanfang; Zhongshan Ophthalmic Centre, Sun Yat-sen University<br>Fu, Haoxiang; Zhenjiang District Hospital<br>Li, Dongfeng; Queen's University Belfast<br>Congdon, Nathan; Queen's University Belfast; Sun Yat-Sen University

**Primary Subject Heading**:	Health economics
Secondary Subject Heading:	Epidemiology, Health services research, Ophthalmology, Public health
Keywords:	Diabetic retinopathy < DIABETES & ENDOCRINOLOGY, EPIDEMIOLOGY, HEALTH ECONOMICS, Health economics < HEALTH SERVICES ADMINISTRATION & MANAGEMENT

SCHOLARONE™
Manuscripts

I, the Submitting Author has the right to grant and does grant on behalf of all authors of the Work (as defined in the below author licence), an exclusive licence and/or a non-exclusive licence for contributions from authors who are: i) UK Crown employees; ii) where BMJ has agreed a CC-BY licence shall apply, and/or iii) in accordance with the terms applicable for US Federal Government officers or employees acting as part of their official duties; on a worldwide, perpetual, irrevocable, royalty-free basis to BMJ Publishing Group Ltd ("BMJ") its licensees and where the relevant Journal is co-owned by BMJ to the co-owners of the Journal, to publish the Work in this journal and any other BMJ products and to exploit all rights, as set out in our licence.

The Submitting Author accepts and understands that any supply made under these terms is made by BMJ to the Submitting Author unless you are acting as an employee on behalf of your employer or a postgraduate student of an affiliated institution which is paying any applicable article publishing charge ("APC") for Open Access articles. Where the Submitting Author wishes to make the Work available on an Open Access basis (and intends to pay the relevant APC), the terms of reuse of such Open Access shall be governed by a Creative Commons licence – details of these licences and which Creative Commons licence will apply to this Work are set out in our licence referred to above.

Other than as permitted in any relevant BMJ Author's Self Archiving Policies, I confirm this Work has not been accepted for publication elsewhere, is not being considered for publication elsewhere and does not duplicate material already published. I confirm all authors consent to publication of this Work and authorise the granting of this licence.

For peer review only

Willingness to pay for diabetic retinopathy screening in Qujiang District, rural Guangdong, southern China: a cross-sectional study

Baixiang Xiao[1, 2], Xuejun Gu[1], Ling Jin[3], Ving Fai Chan[2,4], Yanping Li[1], Carlos Price-Sanchez[2], Yuanping Liu[3], Yanfang Wang[3], Haoxiang Fu[5], Dongfeng Li[2], Nathan Congdon[2,3,6]

1. Affiliated Eye Hospital of Nanchang University, Nanchang City, China
2. Centre for Public Health, Queen's University Belfast, Belfast, UK
3. The State Key Laboratory of Ophthalmology, Zhongshan Ophthalmic Center, Sun Yat-sen University, Guangzhou City, China
4. College of Health Sciences, University of KwaZulu Natal. Durban. South Africa
5. Zhenjiang District Hospital, Shaoguan City, Guangdong Province, China
6. Orbis International, NY, USA

Correspondence to:
Prof Nathan Congdon
Centre for Public Health, Royal Victoria Hospital, Queen's University Belfast, 274 Grosvenor Rd, Belfast BT12 6BA, UK and State Key Laboratory of Ophthalmology, Zhongshan Ophthalmic Center, Sun Yat-sen University, 54 S. Xianlie Road, Guangzhou, China
Email: ncongdon1@gmail.com

Keywords: willingness to pay, diabetic retinopathy, screening, rural, China

Word count: 3458

Running head: Willingness to pay for diabetic retinopathy screening

For peer review only

Abstract

Objective: To determine willingness to pay for a diabetic retinopathy screening (DRS), and its determinants, among people with diabetes mellitus (DM) in Qujiang District of Shaoguan City, rural Guangdong, southern China.

Design: This cross-sectional study was conducted through a large-scale screening program in 2019. We randomly selected 575 (21.5%) among 2,677 people over 18 years old with known diabetes who attended the screening. Participants elected to pay nothing or RMB10–120 (USD 1.6 – 18.8), in RMB 10 intervals, displayed on printed cards. One trained interviewer collected all the data.

Setting: Ten primary health centres in Qujiang District of Shaoguan City, Guangdong.

Participants: 545 from the 575 randomly selected people (94.8%) agreed to participate to the study.

Outcome measures: Proportion of participants willing to pay anything for screening, mean amount they were willing to pay, and determinants of these figures.

Results: Among 545 participants (mean age 64.6 years [SD±10.4], 40.7% men), 327 (60.0%) were willing to pay something for screening, of whom 273 (83.5%) would pay RMB10 – 30 (USD1.6 – 4.7). People living in rural areas and those from lower-income families were more likely to be willing to pay anything, while men, urban residents, and those covered by employer-linked insurance were willing to pay larger sums ($p<0.05$ for all).

Conclusion: Nearly two-thirds of participants were willing to pay for screening in this screening program organized at the primary care level in rural China. This finding offers the potential that such activities can be sustained and scaled up through user fees.

For peer review only

Strengths and limitations of this study

- The study was nested in a large-scale, real-world screening program in rural China, an area where little is known about willingness to pay for diabetic eye screening services.
- The use of payment cards of differing amounts, presented simultaneously to participants for selection, avoided starting point bias in bidding for payment.
- The upper and lower limits of amounts the study offered for participants' selection were based on the actual cost of retinal detection in hospital and in the community.
- The main weakness is that study was conducted at a single location and can only be applied to other settings with caution.

For peer review only

INTRODUCTION

The global number of people living with diabetes mellitus (PwDM) has increased steadily from 108 million in 1980 to 425 million in 2017[1]. It is expected to increase to 693 million cases by 2045[2]. The increasing burden of PwDM in China has followed international trends, with the prevalence of adult PwDM increasing from approximately 2% in the 1980s to over 10% in 2017[3-5]. This dramatic rise in PwDM is due to increases in life expectancy, wider adoption of western diets, sedentary lifestyles and expanding urbanization[5].

While diabetes mellitus (DM) itself promises to grow as a health concern, complications associated with its progression pose additional risks to health. DM can lead to ocular complications requiring further ophthalmological care, straining the global public health system. Of these ocular conditions, Diabetic Retinopathy (DR) is the most common and serious, potentially resulting in irreversible blindness. After 20 years, 90% of persons with Type 1 diabetes and 60% with Type 2 will develop some form of retinopathy. A third of these cases will require treatment to prevent irreversible blindness[6]. Given current numbers of PwDM and the prevalence of DR, the need for laser treatments is estimated to be over 8 million in China at present[5,7].

Interventions at the level of primary care are effective at both reducing the severity and slowing the progression of DR. Methods such as adopting a healthy lifestyle to control blood glucose, adhering to prescribed medicines, undergoing regular retinal examinations, and receiving timely treatment for retinopathy with laser photocoagulation, intravitreal injections, or vitrectomy for more serious cases, have together been proven to reduce blindness from DR by over 90%[8,9]. In particular, establishing robust diabetic retinopathy screening (DRS) protocols

For peer review only

at the level of primary care is a key step in preventing the progression of DR towards visual impairment (VI) or blindness (BL).

Despite the efficacy of DRS in preventing vision impairment, increasing the uptake of DRS has been a significant challenge for many health systems[10,11]. Common barriers to uptake include: a lack of perceived need, patient fear of positive diagnosis, high cost, lack of access, and low quality of service[10]. Additionally, DR is often asymptomatic before the disease affects the macula or causes a vitreous haemorrhage and begins to impair visual acuity. Therefore, many unscreened patients are unaware they have the condition before it becomes severe[12-17]. As DR becomes more prevalent, integrating DRS at all levels of service has never been more important, but its benefits may not be obvious to asymptomatic patients.

As part of a pilot program to develop and enhance primary and secondary healthcare models for DRS, Orbis International and the World Diabetes Foundation (WDF) have supported eye care services for PwDM at 6 county hospitals in Guangdong Province since 2017. The current willingness to pay (WTP) study was conducted as part of the DRS program in Qujiang District of Shaoguan City, northern Guangdong Province, China in the second half of 2019. Though prior studies in the area have examined willingness to pay (WTP) for cataract surgery[18], at present, no such studies on DRS have been completed in China. This study aims to fill this gap by exploring WTP for DRS services in rural North Guangdong Province. Despite the current lack of targeted funding for DRS in most areas of China, charging for this crucial service might be sufficient to sustain the service if beneficiaries are willing to pay enough. The findings in this report will provide context for future decisions regarding providing care for PwDM and establishing community DRS services in rural China.

For peer review only

METHODS

Study design

This cross-sectional study was conducted through a large-scale screening program in 2019. Ethical approval for the DRS study in Qujiang was obtained from Zhongshan Ophthalmic Center, Sun Yat-sen University (ZOC) (approval number: 2019KYPJ067). Written consent was obtained from each participant. PwDM found with sight-threatening DR (R2 or above in the UKNHS DRS system[19]), operable cataracts, or other suspected eye diseases were referred to secondary-level hospitals for further examination or treatment. The study fulfilled the tenets of the Declaration of Helsinki.

Setting and participants

Qujiang is a secondary-level district in North Guangdong province of China, home to 320,000 people in 2018 (a population density of 192 people per km^2), with a 2018 per capita gross domestic product (GDP) of USD7,160.32. Qujiang's GDP is significantly less than the national average per capita GDP of USD10,253.97 in the same year. These figures are typical for other poor rural areas in southern China.

Shaoguan City Railway Hospital and Qujiang County Hospital are secondary-level hospitals in Zhenjiang and Qujiang Districts respectively. Two doctors from the eye department of Shaoguan City Railway hospital were trained in detecting and treating DR with fundus laser and intra-vitreous injection. Trainers for DRS and managers were also trained in the program to arrange periodic, small-scale DRS in rural township health facilities and urban community health centers since 2017. Basic examinations for anterior segment eye disease were provided

For peer review only

at the outpatient department in Qujiang Hospital. Patients suspected of having posterior segment diseases, including DR, would have to travel to the neighbouring district hospital in Zhenjiang or a facility in the city centre.

As in many other places in China, PwDM are encouraged to register with the primary health system in Qujiang District for a free annual health check, monitoring of complications, and health education. Village doctors (VDs) provide free fasting blood glucose checks for people aged over 35 years or anyone wishing to test their glucose levels. People suspected of having DM are referred for further examination at secondary-level hospitals. A total of 3,646 PwDM registered across 10 primary health centres in Qujiang District were informed of these local health bureau-supported DRS services, at which point they were able to schedule appointments with trained VDs.

Study team members at the screening sites included an ophthalmologist, two nurses, one technician, and one interviewer, all trained for the study. PwDM came to the township health units for the screening at a pre-scheduled time.

Of the 3,646 PwDM informed about the screening, 2,677 (73.4%) from across the 10 towns in Qujiang attended. This was the first formally arranged, large-scale DRS conducted in the district. We randomly approached 575 PwDM (21.5%) attending the DRS by generating a random number for the selection of a first participant and then approaching every sixth participant from that point on who arrived for screening. In total, 545 (94.8%) of those approached agreed to participate in the WTP study and were recruited.

For peer review only

Data collection

All participants were informed of the scheduled DRS 1-2 months before screening started, through the trained VDs in the study area and posters circulated in the villages. They were informed of the WTP study by the interviewer at the screening site. Draft questionnaires were tested with some PwDM.

Participants were welcomed to the scheduled annual DR screening by their local public health doctor at each screening site. The local physician would then personally inform each participant when their screening reports were available via mobile phone, text, or WeChat message within one week. Participants were then supplied with DR educational bulletins and posters, which provided information on DR and highlighted the importance of receiving annual fundus imaging for the early detection of ocular diseases.

Participants' basic information and disease history were collected and double-checked via the Chronic Disease Recording System. Retrieved data included participant name, age, sex, site of residence (rural or urban), education, profession, insurance, self-assessment of visual acuity, economic situation including the proportion of family income spent on food, and history of diseases including DM and hypertension. Venous blood was collected from each participant to monitor HbA1c. Presenting visual acuity (PVA) was tested monocularly in each eye at 6 meters with a retro-illuminated Snellen visual chart before the ophthalmologist carried out a slit lamp examination. Intraocular pressure (IOP) was measured in each eye using tono-pen before pupils were dilated with compound tropicamide to enable the capture of fundus images with a Canon CR-2 by a trained nurse. Patients with high IOP or shallow anterior chambers detected under slit lamp examination were not dilated; instead, images were taken through

For peer review only

pupils which had been dilated scotopically after around 3 to 5 minutes in a darkened room.

Diabetic complications were defined as any of the following symptoms: ulcers on hands or feet, cardiovascular disease, diabetic nephropathy, diabetic eye disease, ketoacidosis or other serious metabolic complications. Participants could self-assess their physical health and visual acuity on a scale of 1-5, corresponding to: bad (1), not good (2), alright (3), good (4) and excellent (5). Regarding their economic situation, respondents were given two choices related to the proportion of monthly family income spent on food: less than 30%, equal to or over 30%.

Questionnaires regarding WTP for the DR screening were administered post screening in a quiet, separate room. Each participant was given the opportunity to ask any questions they might have on DM, DR, screening, or the report, and received informed responses from the trained interviewer. Comments on the screening were also invited. The participant was then told that the screening required appropriate human resources, equipment, and preparation, so there was some cost. The direct cost (travel or transport cost, daily allowance for screening staff) of one person screened was approximately USD4.7. At the time of the study, there was no budget in the primary health care system for the DRS. According to the provincial pricing regulations, fundus imaging charges were USD 18.8 for both eyes at secondary level hospitals. Ultimately these costs fall on the patient, as expenses associated with imaging and registration are not currently reimbursed by insurance.

Each participant was informed that their choice of willingness to pay category would not affect their ability to receive primary health care, medical services in hospitals, nor current or any

For peer review only

future DR screening. Participants were first asked whether they would like to pay something, starting at 10 yuan (USD 1.6), for the screening. Reasons were elicited for those answering "no". When a participant expressed willingness to pay any amount, they were presented with cards marked from 10 yuan (USD 1.6) to 120 yuan (USD 18.8) at intervals of 10 yuan (USD 1.6) (12 cards in total). To avoid bargaining bias, we presented participants with cards valued at RMB10, 20, 30, 40, 50, 60, 70, 80, 90, 100, 110 and 120 simultaneously for their selection. A trained interviewer collected all data for WTP to maintain the consistency and quality of the interview, although general data were collected by three trained staff.

For peer review only

Statistical analysis

Participants' characteristics were presented as mean (standard deviation, SD) for continuous data with normal distribution or median (inter quartile range, IQR) for data without normal distribution, and frequency (%) for categorical variables. Shapiro–Wilk normality tests and histograms were applied to check the normality of continuous data. Participants' age was categorized by intervals of 10 years. Duration of diabetes was categorized by intervals of 5 years. The cut-offs of 6/60, 6/18 and 6/12 were used to describe visual acuity in the better-seeing eye.

Logistic regression analysis for WTP and linear regression analysis for the different amounts participants would pay were employed to examine the associations between potential factors and outcomes. Univariable and multivariable regression models were fitted. Age, sex and variables with $P<0.20$ in the univariable analyses were included in the multivariable regression model. All statistical analyses were performed using a commercially available software package (Stata 16, Stata Corp, College Station TX, USA).

The reasons given by the participants who would not pay anything for the DRS at community level were listed in text including all the points mentioned.

Patient and public involvement

Participants were not directly involved in designing or implementing the study, although draft questionnaires were tested with some PwDM.

RESULTS

Among 545 participants, the mean age was 64.4±10.4 years (range 35-92 years), and 323 [59.3%] were women. (Table 1) A total of 345 (63.3%) participants lived in urban settings. Almost half (44.6%) of participants had received a primary school education and 54 (9.9%) attended high school or above. All participants had either standard residential medical insurance (n=372, 68.3%) or employment-linked insurance (n=173, 31.7%).

For peer review only

Table 1. Demographic characteristics

Characteristic	Mean (SD)/n (%)
Total	545
**Age (year), mean (SD)**	64.6 (10.4)
<50	48 (8.8)
50-59	131(24.0)
60-69	198(36.3)
70-79	137(25.1)
80+	31(5.7)
**Gender, n (%)**	
Male	222 (40.7)
Female	323 (59.3)
**Duration of DM (year), n (%)**	
<5	279 (51.2)
5-10	130 (23.9)
10-15	73 (13.4)
>15	63 (11.6)
**Diabetic complications present, n (%)**	
Yes	434 (79.6)
No	111 (20.4)
**Residence, n (%)**	
Urban	345 (63.3)
Rural	200 (36.7)
**Education, n (%)**	
Never received formal education	81 (14.9)
Primary school	243 (44.6)
Junior high school	167 (30.6)
High school or above	54 (9.9)
**Presenting visual acuity in better-seeing eye, n (%)**	
>=6/12	435 (79.8)
>/=6/18 to <6/12	72 (13.2)
>/=6/60 to <6/18	32 (5.9)
<6/60	6 (1.1)
**Medical insurance, n (%)**	
Residential medical insurance	372 (68.3)
Employment-linked insurance	173 (31.7)
**Self-assessment of current health situation, n (%)**	
Bad	11 (2.02)
Not good	107 (19.6)
Good	261 (47.9)
Very good	153 (28.1)
Excellent	13 (2.39)
**Self-assessment of current visual acuity, n (%)**	
Bad	12 (2.20)
Not good	101 (18.5)
Good	349 (64.0)
Very good	74 (13.6)
Excellent	9 (1.65)
**Economic situation, n (%)**	
<30% of monthly family income spent on food	108 (19.8)
⩾30% of monthly family income spent on food	437 (80.2)

SD: standard deviation.

For peer review only

Approximately half (51.2%) of participants had been diagnosed with DM within the last 5 years and only 63 (11.6%) had been diagnosed over 15 years prior (Table 1). Most participants (n=435, 79.8%) had presenting VA >= 6/12 in the better-seeing eye. Most participants (n=434, 79.6%) did not report any diabetic complications. Nearly 80% of participants reported being in good, very good, or excellent health at the time of the screening, and a similar proportion described their vision as falling in the same three categories. Regarding their economic situation, most participants (80.2%) reported their households spent 30% or above of their monthly family income on food, while 19.8% spent less than 30%. (Table 1)

Overall, 327 (60.0%) participants expressed a willingness to pay something for the DRS at the primary level, of whom 273 (83.5%) would pay RMB10 – 30 (USD 1.6 – 4.7), 34 (10.4%) would pay RMB 40 – 50 (USD 6.3 – 7.8), and 15 (4.6%) would pay RMB 80 -120 (USD 12.5 – 18.8). In logistic regression models, only rural residence and those paying >= 30% of income for food (both more willing) were associated with willingness to pay something for DRS (Table 2). Both variables were significant in the univariate and multivariate models.

Among 327 PwDM who were willing to pay something for screening, in univariate linear regression models, men, urban residents, and those with higher educational levels and covered by employment-linked insurance were willing to pay more (Table 3). Conversely, participants with worse self-rated health and vision were willing to pay less. Only the associations with male sex, urban residence and employment-linked health insurance remained significant in the multivariate models. The level of HbA1C were not found having significant association with willingness to pay for DRS.

For peer review only

Table 2. Logistic regression analysis of willingness to pay for diabetic retinopathy screening, Qujiang, China (n=545)

Characteristic	Univariable regression		Multivariable regression* (n=545)	
	OR (95% CI)	P	OR (95% CI)	P
Age (per year)	0.99 (0.97, 1.01)	0.217	1.00 (0.98, 1.02)	0.690
Female sex	0.83 (0.59, 1.18)	0.302	0.98 (0.66, 1.44)	0.903
Duration of DM (year)				
<5	Reference		Reference	
5-10	0.67 (0.44, 1.03)	0.066	0.71 (0.46, 1.11)	0.138
10-15	0.64 (0.38, 1.08)	0.094	0.75 (0.43, 1.30)	0.306
>15	0.85 (0.48, 1.49)	0.568	1.18 (0.64, 2.19)	0.591
Diabetic Complications	0.89 (0.58, 1.37)	0.602		
Urban residence	0.46 (0.31, 0.66)	<0.001	0.44 (0.28, 0.69)	<0.001
Education				
Never received formal education	Reference		Reference	
Primary school	1.07 (0.64, 1.78)	0.795	1.31 (0.75, 2.28	0.350
Junior high school	1.16 (0.68, 1.99)	0.579	1.39 (0.74, 2.63	0.310
High school or above	1.81 (0.87, 3.75)	0.113	2.07 (0.90, 4.78	0.087
Presenting visual acuity in better-seeing eye				
>/=6/12	Reference			
>/=6/18 to <6/12	0.93 (0.51, 1.71)	0.823		
>/=6/60 to <6/18	1.03 (0.58, 1.82)	0.918		
<6/60	1.33 (0.24, 7.36)	0.741		
Standard residential health insurance	1.41 (0.98, 2.03)	0.066	0.94 (0.59, 1.51)	0.804
Self-assessment of health				
Bad	Reference			
Not good	0.96 (0.26, 3.47)	0.947		
Good	0.70 (0.20, 2.46)	0.582		
Very good	1.02 (0.29, 3.63)	0.978		
Excellent	3.14 (0.45, 22.0)	0.248		
Self-assessment of visual acuity				
Bad	Reference		Reference	
Not good	1.00 (0.30, 3.38)	0.996	1.09 (0.30, 3.92)	0.894
Good	0.96 (0.30, 3.08)	0.944	0.74 (0.21, 2.55)	0.629

For peer review only

Very good	1.80 (0.51, 6.32)	0.357	1.13 (0.29, 4.37)	0.854
Excellent	5.71 (0.53, 61.4)	0.150	4.03 (0.34, 47.5)	0.268
Economic situation				
<30% monthly income on food	Reference		Reference	
⩾30% on food	1.66 (1.09, 2.54)	0.018	1.70 (1.04, 2.77)	0.035

OR: odds ratio; CI: confidence interval.

*Age, sex and variables with P<0.20 in the univariable regression analysis were included in the multivariable regression model.

For peer review only

Table 3. Linear regression analysis of participant willing to pay something for diabetic retinopathy screening, Qujiang, China (N=327)*

Characteristic	Univariable regression		Multivariable regression† (n=327)	
	□ (95% CI)	P	□ (95% CI)	P
Age (per year)	-0.0003 (-0.003, 0.002)	0.847	-0.002 (-0.004, 0.001)	0.281
Female sex	-0.10 (-0.16, -0.05)	<0.001	-0.07 (-0.13, -0.02)	0.013
Duration of DM (year)				
<5	Reference			
5-10	0.02 (-0.05, 0.09)	0.628		
10-15	0.006 (-0.08, 0.10)	0.890		
>15	0.05 (-0.04, 0.14)	0.283		
Diabetic Complications Present	0.04 (-0.03, 0.11)	0.300		
Urban residence	0.16 (0.10, 0.21)	<0.001	0.14 (0.07, 0.20)	<0.001
Education				
Never received formal education	Reference		Reference	
Primary school	0.15 (0.06, 0.23)	0.001	0.06 (-0.02, 0.14)	0.162
Junior high school	0.13 (0.04, 0.22)	0.005	0.0001 (-0.10, 0.10)	0.998
High school or above	0.22 (0.11, 0.33)	<0.001	0.09 (-0.03, 0.20)	0.149
Presenting visual acuity in better-seeing eye				
>/=6/12	Reference		Reference	
>/=6/18 to <6/12	-0.04 (-0.14, 0.07)	0.496	-0.005 (-0.10, 0.09)	0.911
>/=6/60 to <6/18	-0.08 (-0.18, 0.01)	0.080	-0.02 (-0.11, 0.07)	0.707
<6/60	0.05 (-0.21, 0.30)	0.718	0.17 (-0.08, 0.42)	0.173
Standard residential health insurance	-0.15 (-0.21, -0.09)	<0.001	-0.08 (-0.15, -0.004)	0.040
Self-assessment of health				
Bad	Reference		Reference	
Not good	-0.27 (-0.47, -0.07)	0.009	-0.13 (-0.47, 0.21)	0.455
Good	-0.22 (-0.42, -0.02)	0.028	-0.07 (-0.43, 0.28)	0.696
Very good	-0.26 (-0.46, -0.06)	0.010	-0.08 (-0.43, 0.28)	0.672
Excellent	-0.22 (-0.47, 0.02)	0.073	-0.32 (-0.73, 0.10)	0.134
Self-assessment of VA				
Bad	Reference		Reference	
Not good	-0.28 (-0.48, -0.08)	0.007	-0.11 (-0.46, 0.24)	0.532
Good	-0.23 (-0.43, -0.04)	0.018	-0.05 (-0.41, 0.31)	0.781
Very good	-0.27 (-0.47, -0.07)	0.008	-0.06 (-0.42, 0.31)	0.756
Excellent	-0.03 (-0.29, 0.23)	0.835	0.33 (-0.10, 0.76)	0.135
Economic situation				
<30% income paid for food	Reference			
≥30% income paid for food	0.007 (-0.07, 0.08)	0.847		

□: parameter coefficient estimate; CI: confidence interval.

*The amount willing to pay was not normally distributed and logarithmic transformation was applied.

†Age, sex and variables with P<0.20 in the univariable regression analysis were included in the multivariable regression model.

Reasons given by participants unwilling to pay anything for the DRS included: should be covered by government or medical insurance; screening report was not received so cannot tell the quality of this service; suspicious whether the money charged would be used properly; suspicious whether the program would be sustainable; would rather go to hospital.

For peer review only

DISCUSSION

In this study, among 545 randomly selected people with diagnosed diabetes who attended a large scale, primary care diabetic retinopathy screening in rural southern China, nearly two-thirds (60%) were willing to pay something for diabetic retinopathy screening. The groups most likely to be willing to pay something were rural-dwellers and economically less-advantaged families. Among participants willing to pay something, those willing to pay more included men, urban-dwellers, and those covered by employment-linked insurance. This study has highlighted a considerable willingness to pay for these services among primary care patients.

The other WTP study for eye health services we could identify in China, conducted in 2006 on cataract surgeries, established that 73% of cataract patients were willing to pay something, and that many were willing to pay more for experienced surgeons and higher-quality intra-ocular lenses[20]. Possible reasons for a lower proportion willing to pay anything for DRS as compared to cataract might include the belief that DRS is a public health service and therefore should be paid for by the government; cataract surgeries are in most cases associated with significant improvements in vision; and coverage rates of medical insurance have increased in China since 2008, potentially reducing willingness to pay out of pocket for healthcare services.

Many countries have experimented with DR screening models to reduce barriers to access and increase service uptake. For example, the United Kingdom and some other European countries have established free community screening for DR[21,22] and found it to be cost-effective[23]. Tele-ophthalmology and general practitioners have been used in the United States, with services either reimbursed by insurance, or paid fully or in part out-of-pocket[24]. General practitioners'

For peer review only

referrals for those detected at DR screenings to retinal specialists for further treatment were shown to be effective in preventing VI from DR in Australia[25,26]. There are also established DR screening services in Asian countries such as Singapore[27], India[27,28] and Taiwan[29].

This study suggests that rural residents and those who are less economically advantaged are more willing to pay for DRS services. In Qujiang District, Shaoguan City, rural residents with DM would otherwise have to travel to the neighboring district hospital or the city centre to seek eye services. Given that there is no public transportation, this trip would take over an hour. Traveling by motorcycle was the most common mode used by rural residents, making an hour-long trip difficult. Additionally, many elderly could only be taken to the city or the neighboring district with the help of a younger person. The fact that this program increased ease of access by bringing DRS services into rural areas may explain why rural residents were more likely to be willing to pay for screening at their township health unit.

Our study found that people covered by employment-linked insurance were more willing to pay higher fees for DRS. This is likely due to their having comparatively better medical insurance and possibly higher incomes. Unfortunately, it was not possible to collect information on income to test this hypothesis in the current study.

In the current study, men and urban residents were willing to pay more for DRS. This could potentially be explained by their being more economically advantaged on average, or their having greater awareness of the importance of DRS. Urban residents may be more apt to understand the need for examinations to prevent diabetic complications and this suggests that future health education interventions should target rural residents, especially rural women.

For peer review only

Another study has reported that more severe DR among persons with type 2 diabetes was associated with lower perceived utility from the patient's preference-based viewpoint[30]. We did not analyse this metric as the number of patients at R3 was not great enough (9 people, 1.7% among the 545 participants). Rather, we compared the self-assessment of visual acuity and WTP, but found no association between WTP and self-assessed health or vision.

Strengths of the current study include its relevance to actual screening activities, having been community-based, and incorporated into a scheduled, large-scale DR screening program supported by the local hospital, health authorities, and primary level health staff. Additionally, to determine the sum participants were willing to pay, we used a direct method with payment cards of differing amounts which were presented simultaneously to participants for selection. This avoided starting point bias in bidding for payment. To avoid range bias[31], we used 10 yuan (USD 1.6) for a small-scale stable range which would not greatly affect the payment value. The minimum value of 10 yuan is equal to the common consultant fee for a hospital outpatient department, and the maximum value of 120 yuan (USD 18.8) is equal to the sum charged for two-eye fundus images taken at secondary level hospitals. The presented value on payment cards was empirical and conceptual, taking account of the actual pricing of ocular medical services as well as the inherent monetary value of 10 yuan (USD 1.6).

Some weaknesses must also be acknowledged. The study was conducted at a single point in time and cannot reflect long-term WTP values as the economy is constantly developing. The study also took place in one mostly rural region, so it does not represent other areas in China. This WTP study is hypothetical and assumes that screening is beneficial to the health of

For peer review only

participants. Cost of screening was introduced and reinforced by eliciting payment amounts. We have also assumed that the respondents were willing to accept the screening as a benefit to their health. The amounts which participants reported they were willing to pay and the amounts they would actually pay may not be the same[31].

To our knowledge, this is the first study on WTP for DRS in China. Cost has been highlighted as the major barrier to establishing DRS at the primary level in China, outside of wealthy areas such as Shanghai, where DRS is well established and fully covered by the metropolitan city government. Findings from this WTP study provide evidence that substantial screening costs could potentially be covered for by patients.

While a considerable percentage of interviewed PwDM demonstrated willingness to take on some personal cost for DRS, studies have noted that organised screening programs result in substantial cost savings for national health systems[19,30]. Recognising that some 50% of current national medical expenditures in China are covered by government medical insurance, investment to establish systematic DRS at the primary care level could substantially reduce costs for the national healthcare system, as well as reducing out-of-pocket medical expenditures[19]. Conducting additional studies in urban areas, as well as in other diverse settings, would contribute to a more complete picture of WTP for DRS in China and help guide the establishment of organised DRS programs. To all the stakeholders of this project, sustainability of the current program would take a key role to serve PwDM in the areas.

CONCLUSIONS

Nearly two-thirds of participants were willing to pay for screening in this screening program

For peer review only

event organized at the care primary level in rural China, which offers the potential that such activities can be sustained and scaled up through user fees.

For peer review only

Contributors

Baixiang Xiao: designed and monitored the whole study, initially drafted and revised the manuscript. Ling Jin: Analyzed data. Xuejun Gu, Ving Fai Chan, Yanping Li, Dongfeng Li: critically commented and revised the manuscript. Carlos Price-Sanchez: corrected English and critically commented on the manuscript. Yuanping Liu, Yanfang Wang, Haoxiang Fu: coordinated data collection and commented critically on the manuscript. Nathan Congdon: Guided the study generally and revised the manuscript.

Competing interests

The authors do not have any conflict of interest regarding the study and publication of this article.

Funding

The study was part of "Diabetes Phase II project" funded by Orbis International (reference no. NA), World Diabetes Foundation (WDF15-1290), Shaoguan Rural Health Express Project (reference no. NA) and the Zhenjiang District Hospital (reference no. NA). Prof Nathan Congdon received Wellcome Trust funding.

Data availability statement

The datasets generated and/or analysed during the current study are available from the first and corresponding authors upon reasonable request.

Ethics approval

Ethical approval for this study was obtained from Zhongshan Ophthalmic Center, Sun Yat-sen University (approval number: 2019KYPJ067).

Acknowledgements

For peer review only

The authors acknowledge the doctors and nurses from all the ten township health units involved to liaise with the local patients, screening site coordination. Financial support from Shaoguan Rural Health Express program was crucial to the success of the DRS in Qujiang. The trained screening team including Ms Qiong Wan as the team leader from Shaoguan Railway Hospital (Zhenjiang District Hospital) was greatly appreciated.

References

1. Cho NH, Shaw JE, Karuranga S, et al. IDF Diabetes Atlas: Global estimates of diabetes prevalence for 2017 and projections for 2045. *Diabetes Res Clin Pract* 2018; **138**: 271-81.
2. Ogurtsova K, da Rocha Fernandes JD, Huang Y, et al. IDF Diabetes Atlas: Global estimates for the prevalence of diabetes for 2015 and 2040. *Diabetes Res Clin Pract* 2017; **128**: 40-50.
3. Yang SH, Dou KF, Song WJ. Prevalence of diabetes among men and women in China. *N Engl J Med* 2010; **362**(25): 2425-6; author reply 6.
4. Bragg F, Holmes MV, Iona A, et al. Association Between Diabetes and Cause-Specific Mortality in Rural and Urban Areas of China. *JAMA* 2017; **317**(3): 280-9.
5. Xu Y, Wang L, He J, et al. Prevalence and control of diabetes in Chinese adults. *JAMA* 2013; **310**(9): 948-59.
6. Stratton IM, Kohner EM, Aldington SJ, et al. UKPDS 50: risk factors for incidence and progression of retinopathy in Type II diabetes over 6 years from diagnosis. *Diabetologia* 2001; **44**(2): 156-63.
7. Liu Y, Song Y, Tao L, et al. Prevalence of diabetic retinopathy among 13473 patients with diabetes mellitus in China: a cross-sectional epidemiological survey in six provinces. *BMJ Open* 2017; **7**(1): e013199.
8. Bailey CC, Sparrow JM, Grey RH, Cheng H. The National Diabetic Retinopathy Laser Treatment Audit. II. Proliferative retinopathy. *Eye (Lond)* 1998; **12 (Pt 1)**: 77-84.
9. Kohner EM, Stratton IM, Aldington SJ, Holman RR, Matthews DR, Group UKPDS. Relationship between the severity of retinopathy and progression to photocoagulation in patients with Type 2 diabetes mellitus in the UKPDS (UKPDS 52). *Diabet Med* 2001; **18**(3): 178-84.
10. Mukamel DB, Bresnick GH, Wang Q, Dickey CF. Barriers to compliance with screening guidelines for diabetic retinopathy. *Ophthalmic Epidemiol* 1999; **6**(1): 61-72.
11. Lee PP, Feldman ZW, Ostermann J, Brown DS, Sloan FA. Longitudinal rates of annual eye examinations of persons with diabetes and chronic eye diseases. *Ophthalmology* 2003; **110**(10): 1952-9.
12. Lee SJ, Sicari C, Harper CA, et al. Examination compliance and screening for diabetic retinopathy: a 2-year follow-up study. *Clin Exp Ophthalmol* 2000; **28**(3): 149-52.
13. Schoenfeld ER, Greene JM, Wu SY, Leske MC. Patterns of adherence to diabetes vision care guidelines: baseline findings from the Diabetic Retinopathy Awareness Program. *Ophthalmology* 2001; **108**(3): 563-71.

For peer review only

14. Sloan FA, Brown DS, Carlisle ES, Picone GA, Lee PP. Monitoring visual status: why patients do or do not comply with practice guidelines. *Health Serv Res* 2004; **39**(5): 1429-48.
15. Agarwal S, Mahajan S, Rani PK, et al. How high is the non-response rate of patients referred for eye examination from diabetic screening camps? *Ophthalmic Epidemiol* 2005; **12**(6): 393-4.
16. Lewis K, Patel D, Yorston D, Charteris D. A qualitative study in the United Kingdom of factors influencing attendance by patients with diabetes at ophthalmic outpatient clinics. *Ophthalmic Epidemiol* 2007; **14**(6): 375-80.
17. Thompson AC, Thompson MO, Young DL, et al. Barriers to Follow-Up and Strategies to Improve Adherence to Appointments for Care of Chronic Eye Diseases. *Invest Ophthalmol Vis Sci* 2015; **56**(8): 4324-31.
18. He M, Chan V, Baruwa E, Gilbert D, Frick KD, Congdon N. Willingness to pay for cataract surgery in rural Southern China. *Ophthalmology* 2007; **114**(3): 411-6.
19. Harris M. The NHS Diabetic Eye Screening Programme: New Common Pathway *https://wwwrcophthacuk/wp-content/uploads/2014/08/Focus-Winter-2012pdf* **Accessed March 8th 2022**.
20. Ko F, Frick KD, Tzu J, He M, Congdon N. Willingness to pay for potential enhancements to a low-cost cataract surgical package in rural southern China. *Acta Ophthalmol* 2012; **90**(1): e54-60.
21. Roberts R, Speight L, Lee J, et al. Retinal screening of patients with cystic fibrosis-related diabetes in Wales -- a real eye opener. *J Cyst Fibros* 2015; **14**(2): 282-4.
22. Nagi DK, Gosden C, Walton C, et al. A national survey of the current state of screening services for diabetic retinopathy: ABCD-diabetes UK survey of specialist diabetes services 2006. *Diabet Med* 2009; **26**(12): 1301-5.
23. James M, Turner DA, Broadbent DM, Vora J, Harding SP. Cost effectiveness analysis of screening for sight threatening diabetic eye disease. *BMJ* 2000; **320**(7250): 1627-31.
24. Rathi S, Tsui E, Mehta N, Zahid S, Schuman JS. The Current State of Teleophthalmology in the United States. *Ophthalmology* 2017; **124**(12): 1729-34.
25. Lee SJ, Livingston PM, Harper CA, McCarty CA, Taylor HR, Keeffe JE. Compliance with recommendations from a screening programme for diabetic retinopathy. *Aust N Z J Ophthalmol* 1999; **27**(3-4): 187-9.
26. Lee SJ, McCarty CA, Sicari C, et al. Recruitment methods for community-based screening for diabetic retinopathy. *Ophthalmic Epidemiol* 2000; **7**(3): 209-18.
27. Ting DS, Cheung GC, Wong TY. Diabetic retinopathy: global prevalence, major risk factors, screening practices and public health challenges: a review. *Clin Exp Ophthalmol* 2016; **44**(4): 260-77.
28. Sharma M CA, Pava R, Sharm R, Pratibha G. An integrated, mobile service for diabetic retinopathy in rural India. *Community eye Health/International Centre for Eye Health* 2011; **24**: 17-8.
29. Lin KD, Hsu CC, Ou HY, Wang CY, Chin MC, Shin SJ. Diabetes-related kidney, eye, and foot disease in Taiwan: An analysis of nationwide data from 2005 to 2014. *J Formos Med Assoc* 2019; **118 Suppl 2**: S103-S10.
30. Tung TH, Shih HC, Chen SJ, Chou P, Liu CM, Liu JH. Economic evaluation of screening for diabetic retinopathy among Chinese type 2 diabetics: a community-based study in Kinmen, Taiwan. *J Epidemiol* 2008; **18**(5): 225-33.
31. Klose T. The contingent valuation method in health care. *Health Policy* 1999;

47(2): 97-123.

For peer review only

Supplement 1: Checklist for Reporting of Survey Studies (CROSS)

Section/topic	Item	Item description	Reported on page #
**Title and abstract**			
Title and abstract	1a	State the word "survey" along with a commonly used term in title or abstract to introduce the study's design.	1
	1b	Provide an informative summary in the abstract, covering background, objectives, methods, findings/results, interpretation/discussion, and conclusions.	2
**Introduction**			
Background	2	Provide a background about the rationale of study, what has been previously done, and why this survey is needed.	4
Purpose/aim	3	Identify specific purposes, aims, goals, or objectives of the study.	5-6
**Methods**			
Study design	4	Specify the study design in the methods section with a commonly used term (e.g., cross-sectional or longitudinal).	2
Data collection methods	5a	Describe the questionnaire (e.g., number of sections, number of questions, number and names of instruments used).	8-9
	5b	Describe all questionnaire instruments that were used in the survey to measure particular concepts. Report target population, reported validity and reliability information, scoring/classification procedure, and reference links (if any).	8-10
	5c	Provide information on pretesting of the questionnaire, if performed (in the article or in an online supplement). Report the method of pretesting, number of times questionnaire was pre-tested, number and demographics of participants used for pretesting, and the level of similarity of demographics between pre-testing participants and sample population.	8
	5d	Questionnaire if possible, should be fully provided (in the article, or as appendices or as an online supplement).	
Sample characteristics	6a	Describe the study population (i.e., background, locations, eligibility criteria for participant inclusion in survey, exclusion criteria).	8
	6b	Describe the sampling techniques used (e.g., single stage or multistage sampling, simple random sampling, stratified sampling, cluster sampling, convenience sampling). Specify the locations of sample participants whenever clustered sampling was applied.	7
	6c	Provide information on sample size, along with details of sample size calculation.	7
	6d	Describe how representative the sample is of the study population (or target population if possible), particularly for population-based surveys.	7
Survey	7a	Provide information on modes of questionnaire administration, including the type and number of contacts, the location where the survey was conducted (e.g., outpatient	7-9

For peer review only

administration		room or by use of online tools, such as SurveyMonkey).	
	7b	Provide information of survey's time frame, such as periods of recruitment, exposure, and follow-up days.	5
	7c	Provide information on the entry process:<br>–>For non-web-based surveys, provide approaches to minimize human error in data entry.<br>–>For web-based surveys, provide approaches to prevent "multiple participation" of participants.	7<br>7
Study preparation	8	Describe any preparation process before conducting the survey (e.g., interviewers' training process, advertising the survey).	7-8
Ethical considerations	9a	Provide information on ethical approval for the survey if obtained, including informed consent, institutional review board [IRB] approval, Helsinki declaration, and good clinical practice [GCP] declaration (as appropriate).	6
	9b	Provide information about survey anonymity and confidentiality and describe what mechanisms were used to protect unauthorized access.	
Statistical analysis	10a	Describe statistical methods and analytical approach. Report the statistical software that was used for data analysis.	9-10
	10b	Report any modification of variables used in the analysis, along with reference (if available).	
	10c	Report details about how missing data was handled. Include rate of missing items, missing data mechanism (i.e., missing completely at random [MCAR], missing at random [MAR] or missing not at random [MNAR]) and methods used to deal with missing data (e.g., multiple imputation).	
	10d	State how non-response error was addressed.	
	10e	For longitudinal surveys, state how loss to follow-up was addressed.	
	10f	Indicate whether any methods such as weighting of items or propensity scores have been used to adjust for non-representativeness of the sample.	
	10g	Describe any sensitivity analysis conducted.	
**Results**			
Respondent characteristics	11a	Report numbers of individuals at each stage of the study. Consider using a flow diagram, if possible.	7
	11b	Provide reasons for non-participation at each stage, if possible.	7
	11c	Report response rate, present the definition of response rate or the formula used to calculate response rate.	7

For peer review only

	11d	Provide information to define how unique visitors are determined. Report number of unique visitors along with relevant proportions (e.g., view proportion, participation proportion, completion proportion).	7
Descriptive results	12	Provide characteristics of study participants, as well as information on potential confounders and assessed outcomes.	7
	13a	Give unadjusted estimates and, if applicable, confounder-adjusted estimates along with 95% confidence intervals and p-values.	
Main findings	13b	For multivariable analysis, provide information on the model building process, model fit statistics, and model assumptions (as appropriate).	9-10<br>13
	13c	Provide details about any sensitivity analysis performed. If there are considerable amount of missing data, report sensitivity analyses comparing the results of complete cases with that of the imputed dataset (if possible).	
**Discussion**			
Limitations	14	Discuss the limitations of the study, considering sources of potential biases and imprecisions, such as non-representativeness of sample, study design, important uncontrolled confounders.	19
Interpretations	15	Give a cautious overall interpretation of results, based on potential biases and imprecisions and suggest areas for future research.	19
Generalizability	16	Discuss the external validity of the results.	17
**Other sections**			
Role of funding source	17	State whether any funding organization has had any roles in the survey's design, implementation, and analysis.	
Conflict of interest	18	Declare any potential conflict of interest.	22
Acknowledgements	19	Provide names of organizations/persons that are acknowledged along with their contribution to the research.	22

For peer review only
